# Review of electromagnetic interference shielding materials fabricated by iron ingredients

**DOI:** 10.1039/c9na00108e

**Published:** 2019-04-01

**Authors:** Vineeta Shukla

**Affiliations:** Nuclear Condensed Matter Physics Laboratory, Department of Physics, Indian Institute of Technology Kharagpur-721302 India vineeta@phy.iitkgp.ernet.in +91 9026690597

## Abstract

Iron (Fe) and its counterparts, such as Fe_2_O_3_, Fe_3_O_4_, carbonyl iron and FeO, have attracted the attention of researchers during the past few years due to their bio-compatibility, bio-degradability and diverse applications in the field of medicines, electronics and energy; including water treatment, catalysis and electromagnetic wave interference shielding *etc.* In this review paper, we aimed to explore iron based materials for the prevention of electromagnetic interference (EMI) by means of both reflection and absorption processes, including the standard methods of synthesis of Fe-based materials along with the determination of EMI performance. It is customary that a proper combination of two dielectric-losses, *i.e.* electrical and magnetic losses, give excellent microwave absorption properties. Therefore, we focused on the different strategies of preparation of these iron based composites with dielectric carbon materials, polymers *etc.* Additionally, we explained their positive and negative aspects.

## Abstract 2

Iron with carbonaceous materials, conducting polymers, dielectric materials or insulating polymers is reviewed.

## Graphical abstract

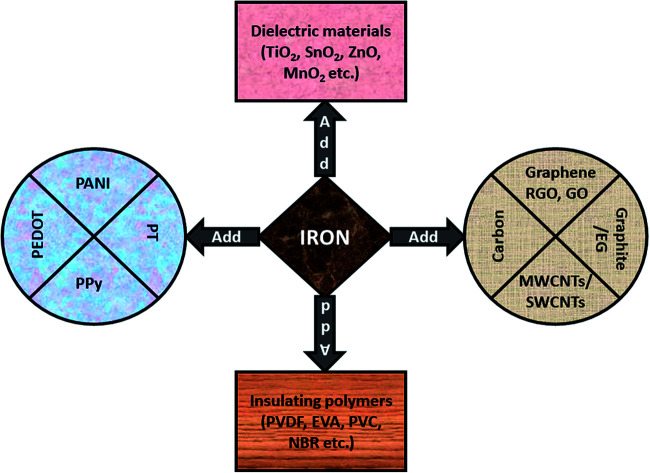

## 1 Introduction

### 1.1 Electromagnetic interference (EMI) pollution

In recent years, electromagnetic (EM) wave radiation in the gigahertz (GHz) range has been regarded as an alarming danger for commercial appliances, biological systems, high quality information technology and defense safety technologies, *etc.*, because when these EM waves interfere with the input signal of the electronic devices, they create a noise that is known as electromagnetic interference (EMI) pollution. In general, EMI pollution could be considered as an undesirable outcome of modern engineering that has become grievous to human health, causing many diseases, *e.g.* headaches, sleeping disorders and trepidation. In communication devices (*e.g.* cell phones, computers, bluetooth devices, laptops), commercial appliances (*i.e.* microwave ovens, the design of microwave circuits) and the automotive industries (*i.e.* integrated electrical circuits), EMI pollution deteriorates the durability and proper functioning of electronic equipment. Therefore, this new kind of pollution has become a serious worldwide problem and its mitigation could be achieved only by use of EMI shielding materials.^1^ EMI shielding is defined in terms of the reflection and/or absorption of electromagnetic radiation by a material that acts as a barrier against the penetration of the radiation passing through the shielding materials. These materials prevent the transmission of EM radiation by reflection and/or absorption of the electromagnetic radiation or by suppressing the EM signals so that EM waves do not affect the functioning and durability of electronic equipment. In general, conductive materials like metals, owing to their high reflectivity, are widely used to isolate spaces or equipment from surrounding EM waves. This reflection shielding is based on the principle of the Faraday cage, in which inside the cage, space is completely impervious to external electric fields. On the other hand, absorption shielding is related to permeable materials *i.e.* magnetic materials. Accordingly, metallic conductors suffer a lack of flexibility, heaviness, and high costs. Meanwhile, ferromagnetic or ferrimagnetic materials have an intrinsic cut-off frequency, usually below the low GHz range, that hinders their use in EMI shielding over a broad GHz range. From this we concluded that, at present, we need to explore broadband shielding materials, those do not only work in the MHz range, but also neutralize EM waves in the GHz range. Most importantly, a lot of effort has been made in this direction; unfortunately to obtain simultaneously minimum reflection with a view to maximum absorption is still challenging task for practical applications.^2–6^

## 2 Scope of review

**Fig. 1:**
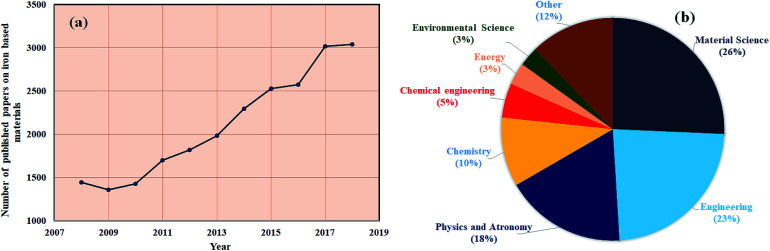
(a) and (b) Scopus database (09/12/2018) for iron based research articles.

Up to date, iron (Fe) based composites have been extensively studied and are most desirable composites in various applications. Ten years of data on Fe-containing composites, collected by Scopus and shown in Fig. 1(a and b), show how the demand for Fe composites has increased year-by-year in several fields of research such as materials science, engineering and many others. It is expected that this review article will benefit ongoing research pertaining to iron nanostructures in the field of EMI shielding, since reviews play a crucial role in continuing interest on current aspects of research in every academic field. Therefore, this review mainly focuses on the development of high performance EMI shielding materials, considering iron as one of the important ingredients.

### 2.1 Mechanisms of shielding

#### 2.1.1 Shielding efficiency in terms of reflection/absorption

**Fig. 2:**
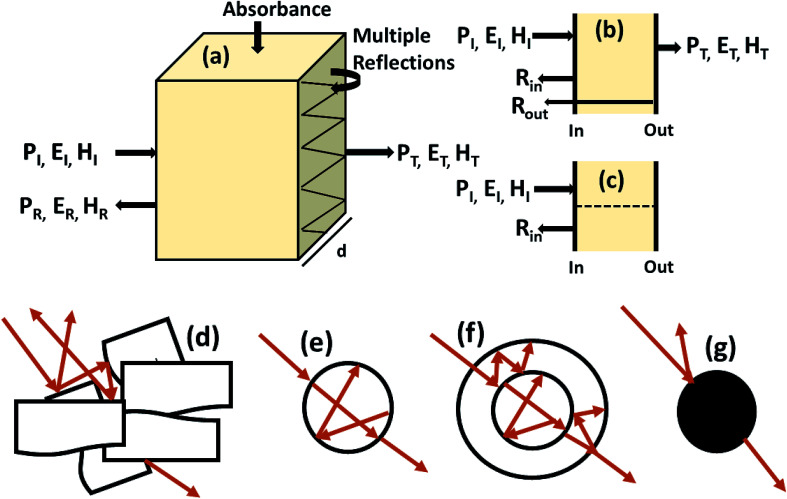
(a) Schematic diagram of incident, reflected and transmitted power and electro-magnetic field intensities when an EM wave is incident on a 3D material; (b and c) sources of reflection in a thin sample (input and output interfaces, *R*_in_ and *R*_out_) and in a thick sample; (d–g) multiple reflections in the case of a porous structure, a hollow structure, a multiple shell structure and a solid sphere.

Shielding efficiency (SE_T_) could be defined as parameter that measures how well a material impedes the EM energy of a certain frequency when passing through it. Fig. 2a represents the possible interactions of EM waves with materials. When the EM waves fall on the front-face of the material then a certain part of the incident power (*P*_I_) is reflected (*P*_R_), while a certain part is absorbed and dissipated in form of energy, and the remaining part is transmitted (*P*_T_) through the shielding material. Therefore, three different processes namely reflection, absorption and multiple internal reflections contribute to the whole attenuation, corresponding to shielding effectiveness SE_R_, SE_A_ and SE_M_, respectively.1
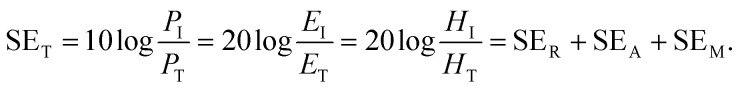
Here *P*, *E* and *H* refer to power and electric and magnetic field intensities while subscripts I, R and T represent the incident, reflected and transmitted components, respectively. Thus, SE_R_ refers to net reflection and SE_A_ represents shielding due to absorption. Note that contributions from secondary reflections (output interface) in Fig. 2a and b occur in finite-dimensional media but in thicker slabs SE_M_ can be neglected (Fig. 2c). Then, the equation takes the form

2 SE_T_ = SE_R_ + SE_A_.

#### 2.1.2 Reflection loss (SE_R_)

The primary mechanism of EMI shielding is reflection. Reflection loss (SE_R_) is related to the relative impedance mismatching between the surface of the shielding material and the EM waves. The magnitude of the reflection loss can be given by3
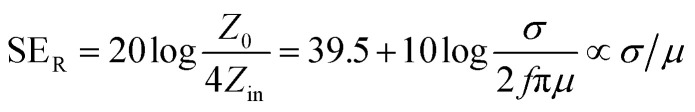
where *σ* is the total conductivity, *f* is the frequency, and *μ* is the relative permeability. It can be seen that SE_R_ is a function of the ratio of conductivity (*σ*) and permeability (*μ*) of the material *i.e.* SE_R_ ∝ (*σ*/*μ*). Thus, for a constant *σ* and *μ*, SE_R_ decreases with frequency. Therefore, materials must have mobile charge carriers (electrons or holes) for reflection of the EM radiation.

#### 2.1.3 Absorption loss (SE_A_)

A secondary mechanism of EMI shielding is absorption. As we know from the plane wave theory, the amplitude of the EM wave decreases exponentially inside the material as it passes through it. Thus, absorption loss results from ohmic losses and heating of the material due to the currents induced in the medium. For conductive materials, absorption loss (SE_A_) in decibels (dB) can be written as:4
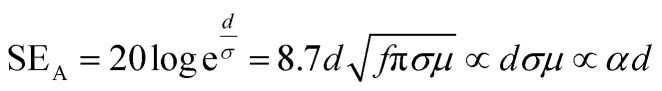
where *d* and *α* are the thickness and attenuation constant of the slab, respectively. The attenuation constant defines the extent at which the intensity of an EM wave is reduced when it passes through the material. It is clear that SE_A_ depends on conductivity (*σ*), permeability (*μ*) and sample thickness (*d*). Such a dependency of SE_R_/SE_A_ on *μ* and *σ* indicates that in magnetic conducting metals, shielding is dominated by absorption rather than reflection. Moreover5
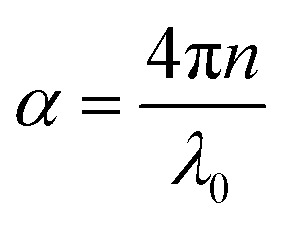
where *λ*_0_ is the wavelength in vacuum and *n* is the refractive index, which is given by (*εμ*)^1/2^; in the case of nonmagnetic materials *μ* = 1. Hence,6
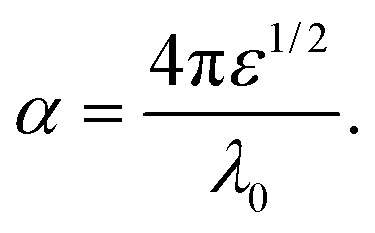


It is clear from eqn (5) that high permittivity is particularly crucial for the enhancement of SE_A_, as well *S*_R_.

#### 2.1.4 Multiple reflection (SE_M_)

For thinner materials, radiation is trapped between two boundaries due to multiple reflection, *i.e.* EM waves reflect from the second boundary, come back to first boundary and are re-reflected from the first to second boundary, and so on, as shown in Fig. 2a.7
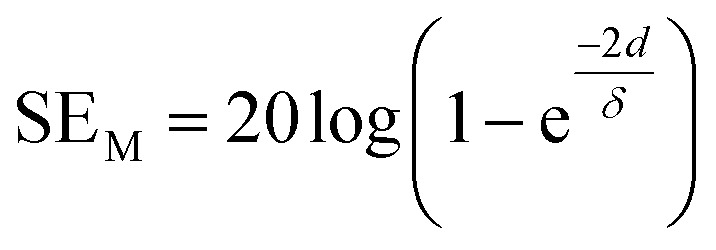
where *δ* is the skin depth, defined as the thickness below the outer surface at which the incident field is attenuated to 1/*e* of its initial value, given by

8 *δ* = (*f*π*σμ*)^−1/2^.

SE_M_ depends on *d* and is closely related to absorption. Hence, multiple reflection plays an important role for porous structures and some definite geometries. For more visualization, Fig. 2d–g shows trapping/scattering of EM radiation by porous, hollow, multi-shell and solid structures. In this structure, a large surface area and a big vacant space excluding the solid structure gives more active sites for scattering and multiple reflection of electromagnetic waves. The hollow/porous structure shows unique properties, *e.g.*, high surface area, disciplinable internal structures, low density and complimentary permeability that can fulfil the quest for improving EMI performance. These multiple reflections (SE_M_) can be neglected when the thickness of the shielding materials is greater than the penetration depth (*δ*) or when SE_A_ is more than 10 dB because in thick shielding materials (high SE_A_) the EM wave hits at the second boundary with negligible amplitude so SE_M_ can be neglected.

#### 2.1.5 Perspective to minimize reflection

It is clear that reflection, SE_R_, depends solely on *σ*/*μ*, while SE_A_ (*dσμ*) also depends on the sample thickness. Such dependency of SE_R_/SE_A_ on *μ* and *σ* indicates that in non-magnetic materials shielding is mainly governed by reflection, while in magnetic conducting metals shielding is dominated by absorption rather than reflection. This situation is quite different for composite materials in which heterogeneous micro structures show the great variations in the local fields due to these nano/micro extent which works as polarization sites. These sites create the lag of the displacement current relative to conduction current. Further, matrix and filler inclusions both have different electro-magnetic properties. In these conditions permittivity and permeability can be replaced by effective permittivity (*ε* = *ε*′ + i*ε*′′) and permeability (*μ* = *μ*′ + j*μ*′′), respectively:

9 *ε* = *ε*′ + i*ε*′′

where i is an imaginary number and *ε* are complex numbers. In the above equation, *ε*′ denotes the electric energy storage capacity, while *ε*′′ is related to dielectric losses. Similarly, permeability is given by

10 *μ* = *μ*′ + j*μ*′′

where j is an imaginary number. In case of magnetic systems, *μ*′ denotes the magnetic energy storage while the imaginary part relates to ohmic losses similar to an electrical system. In complex permeability the *μ*′ and *μ*′′ of the materials are directly related to the energy density and magnetic loss power stored in the magnetic system. Therefore, these possess a complex dependency on the geometry, size, conductivity and volume fraction of each constituent. For several applications such as radar (to reduce the radar cross section) and military applications (*e.g.* hiding military devices), the essential requirement is to adjust the effective permittivity and permeability to certain values by which reflection can be minimized. Therefore, a prerequisite of conductive EMI shielding composites is to limit reflection and enhance absorption for effective EMI shielding materials. This is possible only when we minimize the mismatch of impedance between free space and shielding materials. According to the transmission line theory, intrinsic surface impedance in relation to complex permittivity and permeability for a given medium can be written as,11
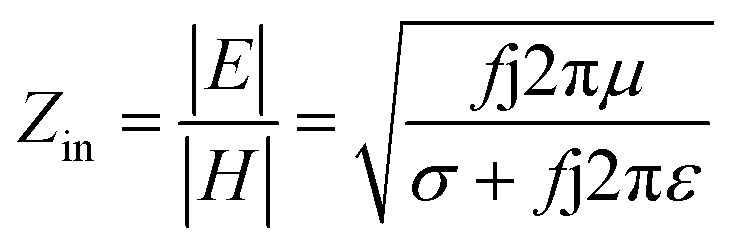


The microwave absorption properties of the materials in terms of reflection loss could be given by12
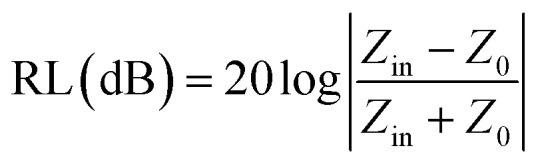


The maximum absorption of microwaves means that a minimum reflection loss (RL_min_) occurs when the impedance of the composite and free space is matched. The ideal impedance matching conditions are when *Z*_in_ = *Z*_0_ = 377 Ω. Here *Z*_0_ is the impedance of air, and *Z*_in_ is the input impedance of the absorber. The above condition is fulfilled at a specific matching thickness (*t*_m_) and matching frequency (*f*_m_). An ideal EM absorption should make the effective width as broad as possible, which can be controlled by the 1/4 wavelength equation:^5^13
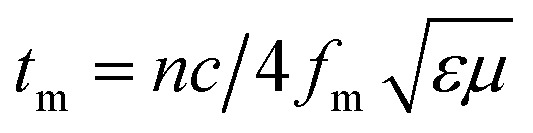
where *n* is the refractive index and *c* is the velocity of light. The RL value of −20 dB is considered to be 99% microwave absorption according to eqn (8) and (11), which is believed as an adequate level of absorption. In order to minimize the impedance mismatch, the best way is to increase the effective permeability or decrease the effective permittivity. Hence, high-performance microwave absorbing materials have been considered extensively to prevent incident EM wave radiation. These materials convert EM energy into thermal energy through dielectric loss and/or magnetic loss by the balance outcome of integralities between the relative permittivity and/or permeability. Moreover, technological fields desire not only efficient shielders, but also fulfil some necessitous criteria such as being lightweight, having a minimum thickness, corrosion- and chemical resistance, good flexibility, tunable morphology, ease of processing, and cheapness.^7^

### 2.2 Factors affecting the EMI performance

#### 2.2.1 Permittivity and permeability

Ideal EMI shielders require impedance matching characteristics of composites which are influenced by permittivity and permeability according to following equation:^8^14
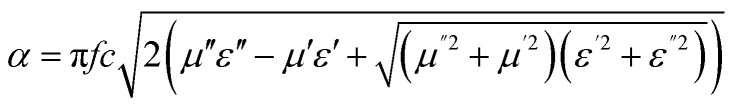
Therefore, permittivity and permeability are crucial parameters to design an effective EMI shielding material, as explained in the previous section. For electrical shielding, conductivity and polarization loss are two key factors that are responsible for the dielectric loss (*ε′′*). Polarization loss could be based on electronic, ionic, dipole orientation (raised by bound charges) and interfacial polarization (due to trapping of space charge). Based on free electron theory, the dielectric loss is given by *ε*′′ = *σ*/2π*ε*_0_*f* or *ε*′′ ∝ *σ*, where *σ* is the conductivity, which indicates that a high electric conductivity enhances *ε*′′. Ionic polarization and electronic polarization works only at the very high frequency region (above 1000 GHz) hence their effects can be neglected in the low microwave frequency region. Dipole polarization comes into the picture due to the presence of defects and residual groups in the material^9,10^ and mainly depends on the fabrication processes, chosen materials, annealing temperature *etc.* The interfacial polarization and respective relaxation appear to be due to trapped space charges at the interfaces. In this case the relaxation process can be investigated by a Cole–Cole semicircle obtained from the Debye dipolar relaxation process. The relationship between *ε*′ and *ε*′′ is

15 (*ε*′ − *ε*_∞_)^2^ + (*ε*′′)^2^ = (*ε*_s_ − *ε*_∞_)^2^

where *ε*_s_ and *ε*_∞_ are the static and relative dielectric permittivity at higher frequencies. If polarization relaxation takes place then an *ε*′′ *versus ε*′ plot will be a single semicircle. This plot is popular as the Cole–Cole semicircle plot. This type of polarization mostly appears in hierarchical and multi-interface composites.

On the other hand, magnetic loss comes from natural ferromagnetic resonance, exchange resonance and eddy current loss in the microwave frequency band. The natural resonance frequency *f*_r_ correlates to an anisotropy field *H*_a_ which can be expressed by the natural-resonance equation: *f*_r_ = *γH*_a_/2π, where *γ*/2π is the gyromagnetic ratio. The anisotropy field *H*_a_ is given by *H*_a_ = 2*K*/*μ*_0_*M*_s_, where *K* is the anisotropy constant and *M*_s_ is the saturation magnetization. A high saturation magnetization (or a smaller anisotropy field) is ascribed to a red shift of the resonance frequency. In other words, a smaller anisotropy field improves the absorption bandwidth. For an excellent microwave-absorbing material, magnetic shielding requires conservation of its magnetic permeability over the GHz range, but it can be seen that at the cut-off frequency *f*_r_, permeability sharply decreases according to the Snoek’s limit, *f*_r_(*μ* − 1) ∝ *M*_s_. Hence, a high *M*_s_ is required at high frequency *f*_r_. Magnetic metals and their alloys (Fe, FeNi, FeCo) possess high *M*_s_ and good permeability, although their high conductive behavior produces eddy current losses resulting in reduced permeability at lower frequencies (in the MHz range). Fortunately, ferrites are semiconducting in nature, but these ferrites possess a significantly lower *M*_s_ value and hence the *f*_r_ occurs at the low GHz range. Therefore, the above-mentioned situations limit their use in the GHz range to the maximum bulk ferromagnetic materials. To overcome the above problem, researchers have focused on nano- or micro-sized materials because these low-dimension materials lower the eddy current loss.

#### 2.2.2 Snoek’s limit

Snoeks limit confers a boundary on the microwave permeability spectrum in magnetic materials. The complex permeability belongs to two type of magnetizing mechanisms: the domain wall and the spin rotation motion, where domain wall and spin rotational term contribution is of the resonance type and relaxation type.^11–13^ Thus permeability is given by

16 *μ*(*ϖ*) = 1 + *χ*_sr_(*ϖ*) + *χ*_dw_(*ϖ*)

where17
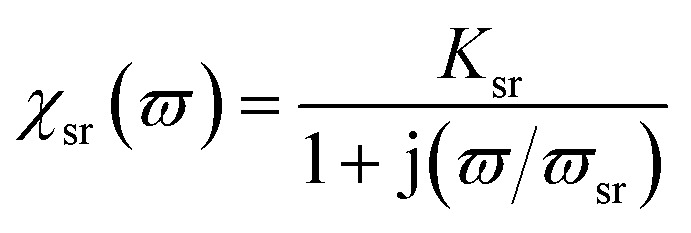
and18
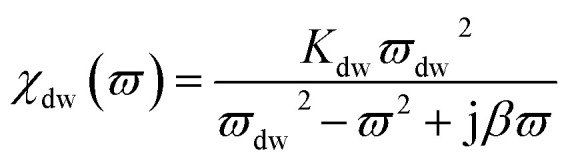
where *ϖ*, *ϖ*_sr_ and *ϖ*_dw_ are the rf magnetic field, the spin resonance and domain wall motion resonance frequencies, respectively. The terms *K*_sr_ and *K*_dw_ define the static spin and domain wall motion susceptibilities while *β* is a damping factor of the domain wall motion. It was observed that only the spin rotational component remains in the higher frequency region; nevertheless the domain wall motion contribution diminishes. Thus at high frequencies (above 100 MHz), complex permeability is governed only by the spin rotational component. In terms of magnetization *K*_sr_ and *ϖ*_sr_ can be written as19
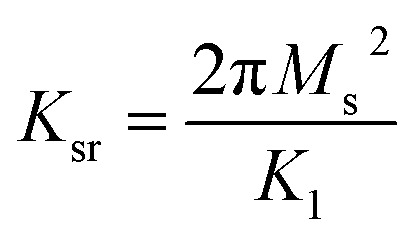
and20
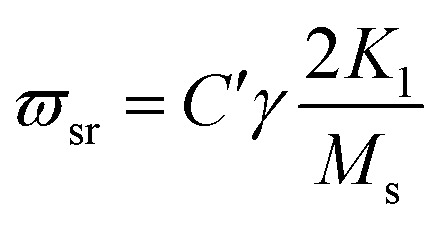
where *M*_s_ is the saturation magnetization, *K*_1_ is the crystalline anisotropy, and *γ* is the gyro magnetic ratio.

21 *ϖ*_sr_*K*_sr_ = 2*C*′π*γM*_s_

At resonance frequency *ϖ*_sr_ = *ϖ*_*r*_ = 2π*f*_r_

22 *ϖ*_r_(*μ* − 1) ∝ *M*_s_

This is called Snoeks limit, which gives a limitation on the permeability in the case of ferrite.

#### 2.2.3 Size, shape and morphology

The dimensions of magnetic particles have a great impact on permeability. It is observed that below a critical small size, eddy current losses decreases due to the decrease in induced eddy voltage (*E*_eddy_ ∝ area). It is believed that anisotropy energy dominates at the small size of the nanostructures due to the breaking of some exchange bonds. The change in anisotropy energy modifies the spin relaxation time or frequency. Apart from the bulk magnet situation, permeability in nanomaterials is governed by relaxation mechanisms, in contrast to the intrinsic resonance which predicts a constant permeability until relaxation. In the superparamagnetic state, spin fluctuation remains very fast due to its small size, hence relaxation occurs at higher frequencies.^7,14–18^ Furthermore, some complicated structures consisting of high porosity and large surface area introduced multi-interfaces that accumulate to bound charges at the interfaces, causing the Maxwell–Wagner effect. In addition, several surfaces within complicated geometries possess unsaturated bonds that are responsible for dipole polarization. Therefore, multi-interfaces are beneficial for electromagnetic attenuation due to conductivity loss and interfacial/dipole orientation polarization. Many Fe and Fe-alloy based systems have been reported that confirm the effect of magnetic anisotropy and relaxation processes.

Bayat *et al.* have observed the effect of particle size and the thickness of material on the EMI performance of Fe_3_O_4_/CFs composites. When the particle size varies from 10–20 nm to 20–30 nm, then SE_Total_ also varies from 47 dB to 68 dB. The above observation shows that larger size particles improve the electrical conductivity as they boost the graphitization of the carbon matrix. Thus, larger Fe_3_O_4_ NPs increase the magnetic permeability of the composite and hence improve the shielding efficiency of the composite. Similarly, thickness variation revealed that a 0.1 mm to 0.7 mm sample thickness enhances SE_T_ from 24 dB to 68 dB. This happened due to an increase in the conductive network, which enhances the SE_A_ and total SE_Total_.

#### 2.2.4 Temperature and time

It is a well known fact that heat treatment increases disorder and creates defects in the form of vacancies, dangling bonds or substitutions in materials, as observed in the ferrite system in which reflection loss is reversed by the annealing temperature.^19^ These defects create an extra energy level around the Fermi level and hence enhance attenuation rather than reflection. Furthermore, reaction time and temperature also influence reflection loss, as reported for FeCo/ZnO composites^20^ because of structural changes that occur as the time and temperature increase.

#### 2.2.5 Mass ratio

Generally, the electrical properties of any of material depend on the percolation threshold value of conductivity:

23 *σ* = *σ*_0_(*V* − *V*_c_)^*c*^

where *σ* is the electrical conductivity of the materials, *σ*_0_ is natural conductivity, *V* is the volume fraction of filler, *V*_c_ is the volume fraction at the percolation threshold and *c* is the critical exponent. At the percolation threshold, conductive networks form within matrices. The percolation threshold depends on certain factors like the shape, morphology, aspect ratio and conductivity of the filler. Moreover, it also depends on the distribution, concentration and compatibility of the filler with the host matrix.^21^ Above the percolation threshold, the properties of the composites start decreasing. For example, in elastomer composites a high volume fraction of filler (mostly metals) in the host matrices decreases the resilience of composites. For this region, a low volume fraction is most desirable. For example, Li and coworkers observed that, in nano Fe_3_O_4_ coated CNTs, reflection loss does not only depend on the Fe_3_O_4_ coating structure, but is also related to the CNT-to-Fe^3+^ mass ratio. This is because the mass ratio ultimately generates dielectric relaxation processes and also enhances the magnetic loss in the form of the eddy current effect.^22^

#### 2.2.6 Thickness

Minimal reflection, RL_min_, of the microwave power occurs when the sample thickness, *t*, of the absorber approximates a quarter of the propagating wavelength multiplied by an odd number, that is24
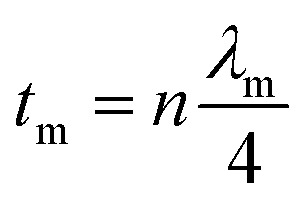
where *n* = (1, 3, 5, 7, 9…), so that *n* = 1 corresponds to the first dip at low frequency. The propagating wavelength in the material (*λ*_m_) is given by25
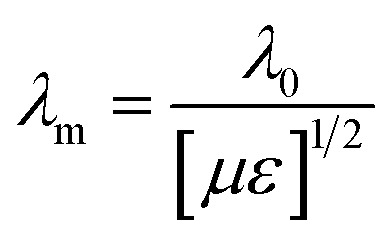


The matching condition results in the cancellation of the incident and reflected waves at the surface of the absorber material, *e.g.* the dips for *t* = 7 mm occurred at the sample thicknesses 1.0(*λ*_m_/4), 3.0(*λ*_m_/4) and so on. Hence, with increasing sample thickness, reflection peaks shift toward the lower frequencies. Apart from sample thickness, coating on the surface of the Fe component also changes the microwave absorption properties. This can be attributed to EM wave dimensional resonance, which increases with the increase of coating thickness. Du *et al.* have shown the influence of shell thickness on the absorption properties of Fe_3_O_4_@C composites. The thickness of the carbon shell in Fe_3_O_4_@C was controlled in the range of 20–70 nm.^23^ A critical thickness of carbon shells shows superior dielectric behavior.

## 3 Measurement techniques

**Fig. 3:**
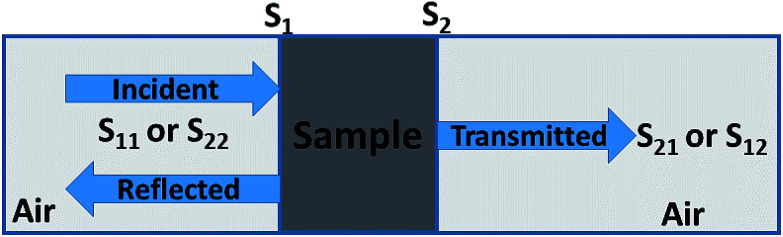
Reflected and transmitted EM wave in a filled transmission line.

Experimentally, network analyzer instruments are used to measure EMI shielding efficiency. There are two types of network analyzer: scalar network analyzers (SNA) and vector network analyzers (VNA). As its name indicates, the SNA measures signal amplitudes only, that is why it is not useful for measuring complex signals. On the other hand, the vector network analyzer (VNA) measures signal magnitude along with various phases. Therefore the VNA is a highly demanded and widely used instrument. In a VNA, its two ports (S_1_, S_2_) indicate the incident and transmitted waves in terms of complex scattering *S* parameters (Fig. 3), *i.e. S*_11_ or *S*_22_ and *S*_21_ or *S*_12_, respectively. These are known as the forward reflection coefficient (*S*_11_), the reverse reflection coefficient (*S*_22_), the forward transmission coefficient (*S*_12_) and the backward transmission coefficient (*S*_21_). Different conversion approaches such as the short circuit line (SCL), NIST iterative, delta-function method, new non-iterative, transmission line theory and Nicolson–Ross–Weir (NRW) technique have been adopted to obtain the characteristic parameters (*i.e. ε*, *μ*, RL and *Z*). The above conversion techniques also have some benefits and limitations. For instance, the short circuit line (SCL) method can estimate *ε* only, while the NIST iterative approach provides *ε* and *μ* but with the limitation *μ* = 1. Among them all, the NRW technique (presented by Nicolson and Ross in 1970 and by Weir in 1974) gives a direct calculation of complex permittivity and permeability from the input *S*-parameters. Therefore, the transmission line theory and the Nicolson and Ross and Weir algorithm are the more popular methods due to their ease of use.^24^ Parameters *Z* (Ω), RL (dB), SE_A_ (dB), SE_T_ (dB) and SE_R_ (dB) can be obtained by using the following equations26
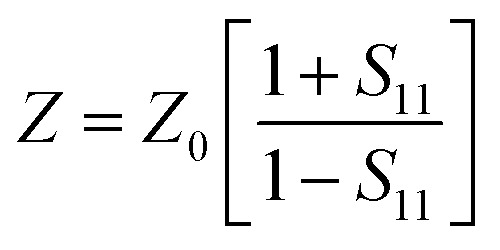

27 RL = 20 log |*S*_11_|

28
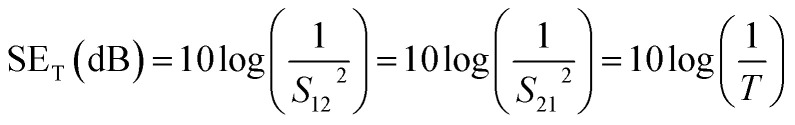
where *T* is the transmittance29
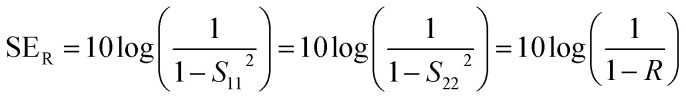
where *R* is the reflectance30
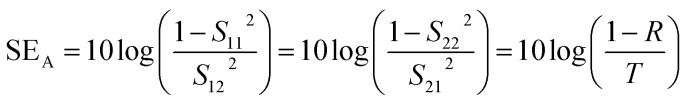
Summation of the reflectance (*R*), transmittance (*T*) and absorbance (*A*) is always equal to 1;

31 *R* + *T* + *A* = 1

Some researchers have also studied impedance matching by means of the delta-function method, in which the delta-function shows the impedance matching degree. The delta-function is given by following equation:^9,25^

32 |*Δ*| = |sin*h*^2^(*Kfd*) − *M*|

where *K* and *M* are33
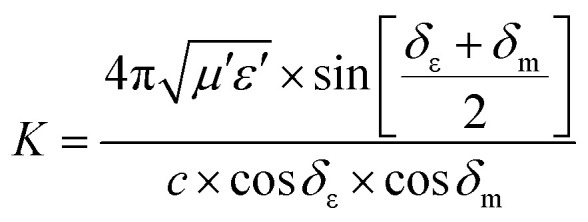
and34
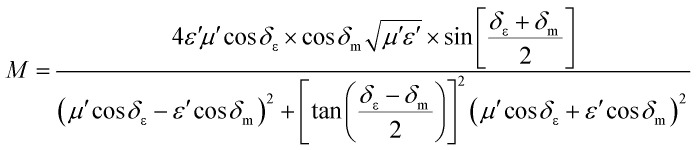
where *δ*_ε_ and *δ*_m_ are the dielectric and magnetic dissipation factor and *c* is the velocity of light. At a certain thickness, the maximum absorption occurs when RL approaches −∞. This leads to better impedance matching when the delta value tends to zero.

## 4 Materials used for EMI shielding

### 4.1 Iron (Fe) ingredient

**Fig. 4:**
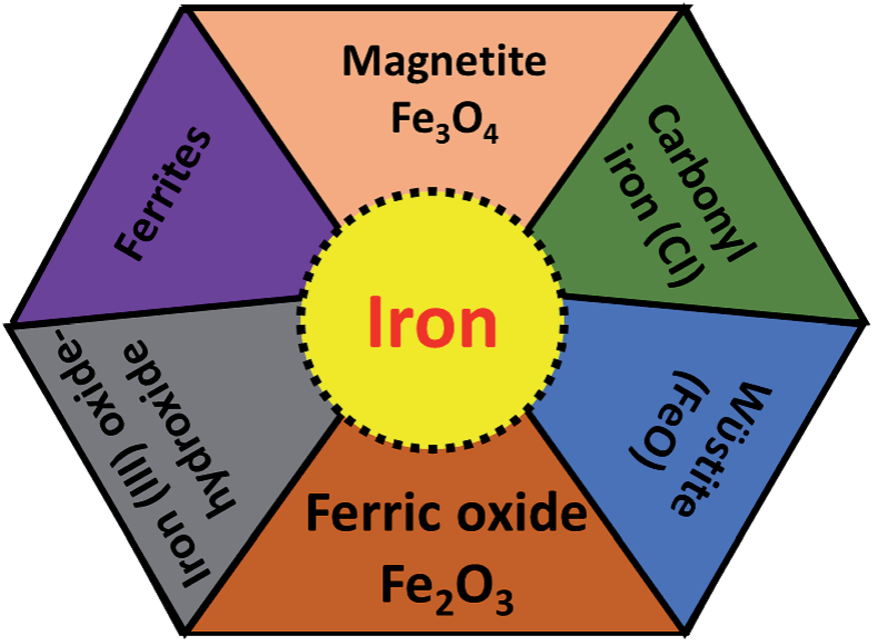
Different type of iron components.

For the development of high performance microwave absorption materials, magnetic nanostructures have been of great interest in the last few years. Their low cost facile synthesis along with the high biodegradability and biocompatibility advantages of iron and other components have made them desirable materials relative to other transition-metals in terms of potential applications. In the earth’s crust, the transition metal iron is the fourth most ubiquitous material that forms the inner as well as the outer surface of the earth. Iron is one of the most promising candidates for several applications including catalysis, microwave absorption, water pollution treatment and magnetic materials and many others. Ion can exhibit from the +2 to the +7 oxidation state, nevertheless the +2 and +3 states are more common due to the ease of hopping of the charge carriers. Fe is well known as the highest room temperature ferromagnetic material with a high saturation magnetization of 218 A m^2^ kg^−1^ at 293 K, and a curie temperature, *T*_C_ = 1043 K, above room temperature. Furthermore, iron is a very soft magnetic material compared to cobalt and possesses low magnetocrystalline anisotropy. For a few decades, design of Fe based nanostructures has increased greatly because nanostructured materials have many advantages such as a high aspect ratio, good porosity and the high magnetic moment (superparamagnetic behavior) of the nanomaterials compared to bulk materials.^26^ Pure Fe is found either in the body-centered cubic (bcc) structure or face-centered cubic (fcc) structures, but exhibits extreme sensitivity of the structure of iron to changes in air conditions (orthorhombic, spinel) and hence the properties (such as electrical, magnetic, optical) of the Fe material. The most common iron species are iron oxides, ferric oxide, magnetite, ferrous oxides (FeO) and iron hydroxide (FeOOH), as depicted in Fig. 4. Although the fabrication of a magnetic iron nanostructure is quite difficult, much effort has been made to prepare Fe nanostructures using ball milling, DC arc plasma and sputtering methods.^27^ Among these, Fe nanostructures such as nanoflakes, nanoparticles and core–shell (Fe as core coated with oxide shell) structures are evidently the more common structures, because oxide shells not only prevent Fe from oxidation in the presence of air, but also prevent the forefront reflections as previously observed in pure Fe sheets that show negligible microwave absorption, due to the good conductivity of Fe elements (*σ* ∼ 10^7^ S cm^−1^) and the strong skin effect at GHz high frequency. This is the main reason that Fe structures have been part of rather few studies. Some other studied Fe-based microwave materials include nanoparticles (NPs) and dendrite-like micro-structures that crystallize in bcc structures prepared by ball milling and hydrothermal process, respectively. The reflection loss of Fe NPs was observed by pelleting in a paraffin matrix, so for Fe/paraffin = 4/1, RL_min_ = 11 dB at 13.6 GHz. A complete energy dissipation of the EM wave occurs means no reflection and satisfies the impedance matching condition *Z* = *Z*_0_, indicating the absence of an actual absorbing resonance. In case of dendrite-like micro-structures, *M*_s_ is found to be higher than Fe nanoparticles but less than the bulk and hence attains an RL_min_ = −25.0 dB (matching frequency 2.5 GHz, matching thickness 3 mm). The excellent microwave absorption properties of Fe dendritic microstructures could be result of their hierarchical morphology, providing surface defects and a large surface area.^22,28–30^ More importantly iron occurs in various shapes, size and dimensions, such as nanowires, nanoparticles, nanorods, nanotubes, hollow fibers, microspheres and dendrite-like microstructures^31^ which enhance the reflection loss, but can moderate the conductivity. To overcome the above problem, iron oxides such as ferric oxide, magnetite and ferrous oxides (FeO) have been preferred for the design of effective microwave absorption materials because they are semiconductors (highly resistive).^32^

#### 4.1.1 Ferrites

Ferrites have iron oxide as their main constituent, along with other metal oxides. These materials have been used from more than half a century due to their interesting magnetic properties. Compared to iron, ferrites possess high resistivity (0.1–10^−5^ Ω-m), high saturation magnetization and a tunable anisotropy field which make them a preferable choice in a wide range of applications such as bubble devices, the memory cores of computers and microwave devices, recording media, magnetic motors *etc.* Depending upon the crystal structure, ferrites can be classified into the following types:

##### 4.1.1.1 Spinel ferrite

Spinel ferrites are given by formula PFe_2_O_4_, where tetrahedral and octahedral interstitial sites are designated with P (divalent metal ions like Cu, Co, Mn, Ni, Zn) and Fe, respectively. Considering its applicability in the microwave region, the spinel ferrites can be utilized as microwave-absorbing materials, because these ferrites have large magnetic losses and moderate conductivity (semiconductor property). However, spinel ferrites in MW-absorbing applications are restricted because of their low natural magnetic resonance frequency.

##### 4.1.1.2 Garnet

This is described by Pe_3_Fe_5_O_12_ where Pe stand for a trivalent ion *e.g.* a rare earth element. These ferrites have a similar structure to spinel ferrites but with some extra sites (a dodecahedral *c* axis). Doping of cations in these sites may be helpful because lattice interaction with these sites may tune the physical properties of ferrites.^33^ Like spinel ferrites, garnet ferrites are soft ferromagnetic materials with high remanence, a large saturation magnetization and low coercivity. Moreover, the good chemical stability and EM compatibility of these ferrites show their potential for EMI suppression.

##### 4.1.1.3 Ortho-ferrites

The general formula for these ferrites is PeFeO_3_, where Pe is a large trivalent metal or rare earth ion such as Bi or Y. These ferrites exhibit a weak/canted antiferromagnetism with affluent magnetic properties. For instance, ortho-ferrite shows a phase transition from paramagnetic to antiferromagnetic at 620–750 K. Moreover, these kind of ferrites possess excellent multi-ferroelectricity and tunable magnetic properties in which the interaction between Fe^3+^ and Pe^3+^ ions decides the magnetic properties of the ferrites.^34^

##### 4.1.1.4 Hexagonal ferrites

Hexagonal ferrites have a high magnetocrystalline anisotropy field and a planar anisotropy that improves their natural resonance in the upper gigahertz range. This property of hexagonal ferrites increases their versatility in a variety of applications. These ferrites crystallize in a hexagonal structure. Apart from spinel ferrites, the magneto-plumbite structure of these ferrites enables theme to working in the entire GHz range due to their high intrinsic magnetocrystalline anisotropy.^35^ Hence, some of them have gained considerable technological importance in recent years. There are six type of hexagonal ferrites:

##### 4.1.1.4.1 M-Type

M-Type ferrites are given by the formula PFe_12_O_19_ where P = Ba, Sr, Mg, Pb *etc.* These ferrites are composed of the form SRS*R*, in which R and S indicate the three and two oxygen-ion layer blocks. The large magneto-crystalline anisotropy, inexpensive price, high Curie temperature and competent saturation magnetization properties of these kind of ferrites stand them as effective microwave materials.

##### 4.1.1.4.2 Y-Type

The Y-type ferrites are ferrimagnetic materials, generally given by the formula P_2_Q_2_Fe_12_O_22_ where P = Ba, Sr, Mg, Pb, and Q = Cu, Co, Zn *etc.* The magnetic properties of these type of ferrites are greatly susceptible to their crystalline structure, especially in presence of a magnetic environment. Thus, the addition of divalent, trivalent and tetravalent species in these hexaferrites controls their magnetic characteristics in order to obtain improved microwave absorption.^36^

##### 4.1.1.4.3 W-Type

W-Type ferrites are given by the formula P_2_Q_2_Fe_16_O_27_. The crystal structures of these ferrites are closely related to the M-type. The characteristics of these ferrites depend on their particle size or morphology, synthesis method and the distribution of the cations in the crystal structure. These hexagonal ferrites are made up of the structure SSRS*S*R* in which R is a three oxygen-ion layer block with a composition of PFe_6_O_11_, S is a two oxygen-ion layer block with the composition of Fe_6_O_8_, called the spinel block. In the above equation, an asterisk indicates the rotation of the block by 180° along the hexagonal axis. The W-type structure composed of spinel blocks is twice as thick with respect to the M-type hexagonal structure.^37,38^

##### 4.1.1.4.4 X-Type

X-Type ferrites are represented by the formula P_2_Q_2_Fe_28_O_46_. These are composed by the structre 3(SRS*S*R*). X-Type hexagonal ferrites can be considered as a mixture of M and W-type hexagonal ferrites. In comparison with M and W-type hexagonal ferrites, these ferrites possess a larger Curie temperature and saturation magnetization, and hence work as excellent microwave absorbing materials.

##### 4.1.1.4.5 Z-Type

The Z-type ferrites are given by the formula P_3_Q_2_Fe_14_O_41_. These hexagonal ferrites have much good permeability and a higher resonance frequency (*f*_r_) in comparison with spinel ferrites. That is why these ferrites are only used in microwave devices like antennas, inductors and absorbers *etc.*^39^

##### 4.1.1.4.6 U-Type

The U-type hexagonal ferrites are represented by the formula P_4_Q_2_Fe_36_O_60_. Among the hexagonal ferrites, the U-type ferrites possess better thermal stability, a large magnetic anisotropy (*H*_a_) and a large saturation magnetization (*M*_s_).^35^ Therefore these ferrites have been used in many studies on EMI applications.

#### 4.1.2 Ferric oxide (Fe_2_O_3_)

**Fig. 5:**
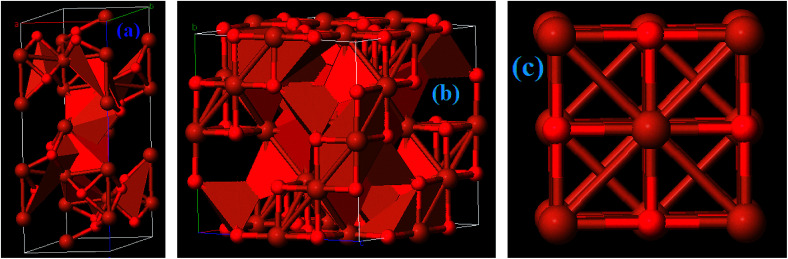
Crystal structure of (a) Fe_2_O_3_, (b) Fe_3_O_4_, and (c) FeO materials.

Among the iron oxides, biocompatible Fe_2_O_3_ is the most common oxide of iron. Therefore, it is one of the most extensively used biomaterials in different applications like cell separation and drug delivery *etc.* Fe_2_O_3_ occurs in an amorphous form and consists of four polymorphs (alpha, beta, gamma and epsilon).^31^ The multitudinous polymorph structures α and γ named as hematite and maghemite, respectively. The α-Fe_2_O_3_ has a rhombohedral–hexagonal type structure, whereas γ-Fe_2_O_3_ shows a cubic spinel structure, as shown in Fig. 5a. On the other hand, the β-Fe_2_O_3_ and ε-Fe_2_O_3_ polymorphs have cubic bixbyite and orthorhombic structures. The α- and β-Fe_2_O_3_ are termed antiferromagnetic and paramagnetic materials, respectively. Hence these are extremely useful in photocatalysis, conversion of pigments, solar energy and water treatment. In contrast, γ and ε-Fe_2_O_3_ possess ferromagnetism^40^ so that these are particularly useful in bio-medicine. α-Fe_2_O_3_ and γ-Fe_2_O_3_ have been widely investigated in EMI shielding applications. Different composites comprising α-Fe_2_O_3_ in attractive morphologies such as popcorn-like α-Fe_2_O_3_, coin-like α-Fe_2_O_3_, watermelon-like α-Fe_2_O_3_ microspheres, α-Fe_2_O_3_ nanorods and hollow γ-Fe_2_O_3_ have been studied and have shown excellent microwave performance. In general, thermo-chemical, two step hydrothermal, solvothermal, chemical reduction and sol–gel approaches are some of the reported methods which have been employed to prepare Fe_2_O_3_ based composites.^41–46^

#### 4.1.3 Magnetite (Fe_3_O_4_)

Among all the Fe oxides, Fe_3_O_4_ is the most comprehensively investigated magnetic nanostructure because of its ease of synthesis, high biocompatibility, superparamagnetic nature, high chemical stability, low toxicity *etc.* Several low cost preparation methods of Fe_3_O_4_ nanostructures can be found in the literature such as sol–gel, solvothermal, co-precipitation and magnetic separation methods *etc.* As a result, magnetite has versatile applications in fields of magnetic storage devices, food analysis, magnetic resonance imaging (MRI), segregation of biomolecules, hyperthermia, and EMI applications,^47^ particularly in the field of magnetism owing to its high magnetic moment. Moreover, Fe_3_O_4_ nanostructures possess a cubic inverse spinel structure with two Fe^3+^ and one Fe^2+^ valence state in which oxygen frames a fcc closed-pack structure, as depicted in Fig. 5b. It is an indispensable kind of half-metallic material in which electron hopping takes place between the Fe^2+^ and Fe^3+^.^48,49^ Consequently, the outstanding magnetic/dielectric properties of the Fe_3_O_4_ nanostructure make it a favorable candidate for magnetic/electric attenuation sources in the EMI shielding mechanism. Fe_3_O_4_ has an abundant number of morphologies *e.g.* it occurs in sandwich-like Fe_3_O_4_, dendritic forms, and as nanorods, nanoparticles, microspheres and nanospindles. Thus, Fe_3_O_4_ can be considered as a good choice for energy applications, including EMI.

#### 4.1.4 Wüstite (FeO)

Iron(ii) oxide (FeO) has a cubic (rock salt) structure in which iron and oxygen atoms are octahedrally coordinated to each other, as depicted in Fig. 5c. FeO is not stable at normal temperature and hence shows high temperature and pressure stability only above 560 °C (ref. 50) which results in high costs of preparation and limits its potential application. Therefore, FeO has rarely been studied. Zhu *et al.* prepared for the first time Fe@FeO dispersions in a polyurethane (PU) matrix.^51^ It was seen that Fe@FeO NPs became magnetically harder after being dispersed in the PU matrix. Fe@FeO/PU possess a significant eddy current effect hence RL is >20 dB even at larger absorber thicknesses. Nevertheless, a coating of SiO_2_ exhibits better performance than Fe@FeO and Fe@FeO/PU composites because the silica shell significantly reduces the eddy current loss and causes an upsurge in the anisotropy energy.

#### 4.1.5 Iron oxy-hydroxide (FeOOH)

Iron(iii) oxy-hydroxide occurs in following forms: goethite (α-FeOOH), akaganeite (β-FeOOH), lepidocrocite (γ-FeOOH) and feroxyhyte (δ-FeOOH). These are widely used in electrode materials and lithium batteries. Iron(iii) oxy-hydroxide has poor magnetic as well as electrical properties, which are a primary requirement for EMI applications. Therefore iron(iii) oxy-hydroxide materials are not very popular among material scientists.

#### 4.1.6 Carbonyl iron (CI)

Finally, carbonyl iron (CI) is another captivating magnetic absorbing material that has attracted much attention due to its virtuous properties including superior saturation magnetization, a high Curie temperature, and a high magnetic loss with low permittivity. Interestingly, the magnetic properties of CI are tunable in accordance with its size, morphology and shape. In fact, planar anisotropy as observed in CI nanoflakes, effectively improves the Snoek’s limit which increases the permeability and resonance frequency at the same time. Besides, in the high frequency range, such flakes-type structures can ignore the skin effect.^52^

Although these Fe materials offer several advantages, their high density, heavy weight, processing difficulties, flexibility and narrow absorption bandwidth impede their further application. Fe materials suffer from the skin depth problem; on the other hand ferrites are restricted by Snoek’s limit. As we explained earlier, to design an excellent microwave absorbing material, one needs to optimize its permeability and permittivity, due to the magnetic/dielectric loss capabilities of EM energy. Hence, poor permittivity in comparison to permeability is the main drawback of these Fe oxides. Accordingly, scientists have mainly concentrated on materials which show the complementary relation between permittivity and permeability. In this direction, conducting polymers and carbon based materials have attracted the attention of researchers. Many strategies have been employed to develop effective shielding materials.

## 5 Anchoring of metal oxides

Anchoring of transition metal oxides such as ZnO, ZrO_2_, MnO_2_, SnO_2_, BaTiO_3_, TiO_2_, SiO_2_ with Fe ingredient enhance the permittivity of EMI preventing materials. Thus the combination of these oxides with Fe ingredients significantly improve the dielectric losses and magnetic losses in materials by mean of double attenuation mechanism which is accountable for superior microwave absorption performance. Their cheap, natural richness and environmentally friendly properties make them more accessible for EMI shielding. To date, numerous Fe and transition metal oxides with great EM properties have been explored. However, these semiconductor oxides are restricted at the high GHz range due to their lack of permittivity. Moreover, processing-related difficulties, agglomeration during synthesis and poor dispersion are major drawbacks in the use of Fe/metal oxides composites.

## 6 Conducting polymers (CPs)

**Fig. 6:**
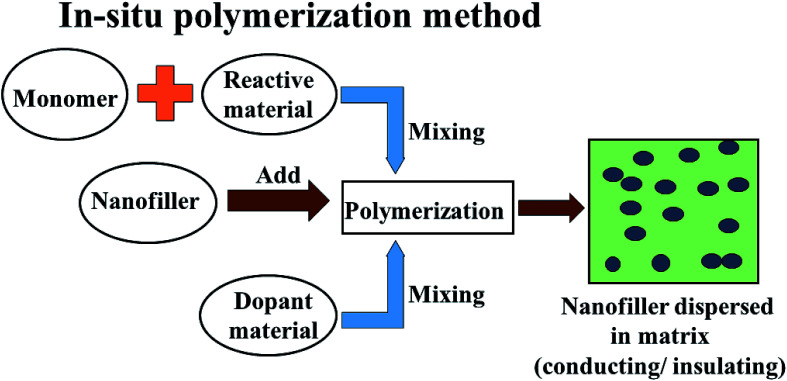
The *in situ* polymerization method of preparation of conductive and insulating polymers.

In comparison to conventional metals and semiconductors, conducting polymers (CPs) possess exclusive properties such as a lower density (1–1.3 g cm^−3^) than iron (7–8 g cm^−3^), gentle processing and preparation conditions, structural flexibility, and most importantly tunable conductivity (0.1–10^−10^ S cm^−1^). Conductive polymers have various applications in sensing, metal corrosion protection, and specifically in energy storage like electromagnetic shielding and microwave absorption. The peculiarities of conducting polymers are believed to depend on their doping level, dopant ion size, water content and protonation level. Two well-known methods have been reported to prepare CPs: CPs are either prepared by electrochemical oxidative polymerization, or by the chemical oxidative polymerization method. Chemical oxidative (*in situ*) polymerization is the most frequently used method to prepare such polymer composites, and is also known as the chemical encapsulation technique. In this method, a filler such as Fe_3_O_4_ nanoparticles are first dispersed in a liquid monomer. The polymerization reaction is initiated by heat/radiation, the diffusion of the appropriate initiator takes place, then the organic initiator/catalyst is set on the surface of the nanoparticles under the required temperature, pressure and stimulation (stirring) conditions, as shown in Fig. 6. In fact, fabrication of polymer nanocomposites is a hybridization process between the organic/inorganic polymer matrix and the inorganic/organic nanofiller to achieve a single material which comprises integrated properties with respect to the matrix and filler only.^53^ This method also helps the modulation of shell thickness in the case of a core–shell structure just by controlling the weight ratio of the monomer and the Fe-based nanostructure, which influences the EM wave absorption properties effectively. According to dissipation mechanisms, microwave absorbing materials show dielectric loss and magnetic loss. In microwave absorbing materials, conducting polymers (CP) serve as dielectric loss materials which makes them the most attractive candidate.^54^ Among the various conducting polymers polyaniline (PANI), polypyrrole (PPy), poly(3,4-ethylenedioxythiophene) (PEDOT), polythiophene (PT), polyfuran (PF), poly(*para*-phenylene) (PPP) and poly(phenylenevinylene) (PPV) are of particular interest due to their easy availability, environmental sustainability, cost-effectiveness and versatile doping chemistry.

### 6.1 Polyaniline (PANI) polymer

Among the different conducting polymers, polyaniline (PANI) is one of the most commonly used polymers as a host material for micro/nano-sized nanofillers owing to its unique physico-chemical properties. These polymers show improved mechanical properties (tensile strength and elongation at break), thermal stability and particularly enhanced electrical conductivity and magnetic properties; these are the prerequisites for the design of effective EMI shielding materials. In comparison with other CPs, PANI is one of the oldest CPs and was first highlighted in 1862 due to the oxidation of an aniline monomer in sulphuric acid. The conductivity of PANI lies between 0.1 and 10^−10^ S cm^−1^. Moreover, PANI is a biocompatible and anti-corrosive polymer which has a controllable dielectric loss ability and is feasible for composition with micro/nano-sized magnetic metals.^55^ Over the last two decades, many efforts have been made to prepare composites comprising polymers and nanofillers. However, improvement of the electric and magnetic properties of the filler/polymer composites are insufficient to design effective EMI shielding materials; one important factor that is still required is how to combine influentially the permeability and permittivity of the these composites. To fulfil these conditions ferromagnetic materials possessing high permeability such as Fe, Fe_3_O_4_ and Fe_2_O_3_ and dielectric materials such as TiO_2_, SiO_2_, and ZnO are widely used in polymer composites. At broad GHz range, however, these dielectrics suffer from a lack of permittivity. For this purpose, carbonaceous materials such as graphene, MWCNT and RGO have also been used with these polymers.

### 6.2 Polypyrrole (PPy) polymer

After the PANI polymer, polypyrrole (PPy) is another most promising conductive polymer because of its tunable stability and ease of preparation, but suffers from poor mechanical strength and processability problems along with insolubility and infusiblity.^56^ These drawbacks hinder its commercial application. To conquer the above problems, magnetic/metal nano-fillers as inorganic filler can be used with PPy to integrate the electro-magnetic properties of polymer composites. These nanoscale fillers have received increased interest due to their intriguing properties arising from their large surface area and nanosize in the host matrix. When the proper combination of magnetic nanofillers along with dielectric materials are encapsulated within the PPy polymer matrix then these polymer composites provide a new perspective to tune the dielectric and permeability properties of magnetic and dielectric materials in a different way by the control of the polymer structure and functionalisation.

### 6.3 Poly(3,4-ethylenedioxythiophene) (PEDOT) polymer

Among the conductive polymers, a polythiophene derivative poly(3,4-ethylenedioxythiophene) (PEDOT) possesses a moderate band gap, controllable electrical conductivity, attractive electrochemical activity. Interestingly, the light weight, easy synthesis processes, good environmental stability, and dielectric loss ability properties of PEDOT make it a promising microwave absorbing material. It is a well known fact that poor EM impedance matching is attributable to magnetic or dielectric loss only. For this purpose, to achieve excellent microwave absorption performance PEDOT has been used with magnetic γ-Fe_2_O_3_, Fe or Fe_3_O_4_ components.^47^

### 6.4 Polythiophene (PT) polymer

Similar to other conducting polymers, polythiophene (PT) is used in anti-corrosion devices, rechargeable batteries and chemical sensors. Similar to other CPs, the conductivity of the PT materials could be controlled from a conducting to an insulating nature by only changing the polymerization route. Nevertheless, its poor solubility restricted its commercial use in many applications. Besides, the inert sulphur atom in thiophene enhances the oxidation potential, which makes the fabrication of polythiophene more complicated.^57^ Therefore, these polymers have been the subject of few studies.

## 7 Nonconducting polymers nanocomposites

**Fig. 7:**
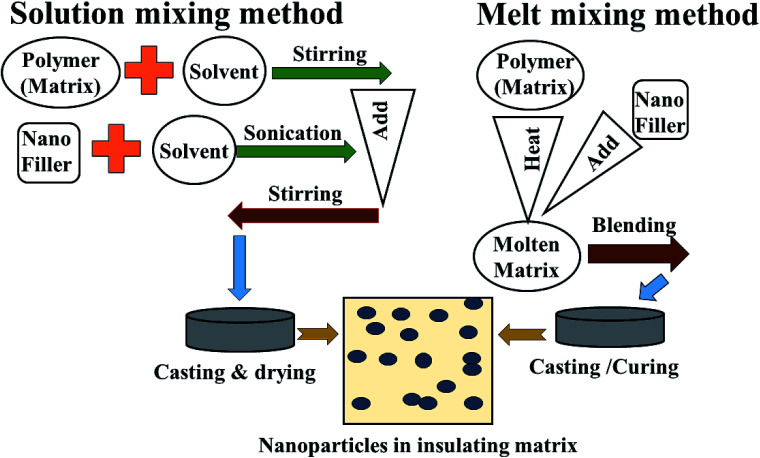
Method of preparation of extrinsic polymers: (1) solution mixing and (2) melt mixing methods.

Though the conductive polymers have many advantages, they suffer from a lack of flexibility and processability during the large scale production of materials. Herein, insulating polymers like rubber and resin have been utilized as alternative substrates for conductive polymers. This is because non-conducting/extrinsic polymer synthesis processes are very cheap, easy, time sparing and environmentally stable. In addition, they can be prepared on large-scale quantities. To overcome the poor electrical, thermal and mechanical properties of these insulating polymers, metal, alloy and carbon nano fillers are often mixed into the polymer matrices to enhance the mechanical strength, conductivity and permeability, which improves reflection well as absorption, depending on the filler characteristics. Mostly facile solution mixing, melt mixing and *in situ* polymerization methods are used for the preparation of these polymer-containing composites. In the solution mixing method the polymer and filler are dissolved or dispersed in a common solvent and undergo a stirring and sonication process until the complete mixing/blending of matrix and filler occurs, followed by casting and drying of the as-prepared composites. In melt blending/mixing, the polymer is melted at high temperature. To avoid the use of a solvent, the mixing of filler and matrix (polymer) takes place at high temperature followed by cooling and drying, as shown Fig. 6 and 7. *In situ* polymerization processes have been generally used to synthesize nanocomposites having insoluble and thermally unsteady matrices (insulating polymers) that can not be developed by solution/melting methods. For more details, some of these extrinsic polymers are explained in later section.

### 7.1 Polyvinylidene fluoride (PVDF) polymer

The fantastic piezoelectric behavior, light weight, compact size, good flexibility, and excellent dielectric properties of the PVDF thermoplastic open up the door to wide applications in various fields. Polyvinylidene fluoride (PVDF) (transparent to light) is a semi-crystalline polymer having significant thermal stability and good chemical resistance among polymers. It occurs in five crystalline phases α, β, γ, δ and ε, each with different chain conformations. Hence, PVDF as a matrix in nanocomposites is one of the key parameters for a wide range of applications. Pure PVDF has poor EMI shielding properties,^6^ but the addition of Fe based nanoparticles within the PVDF matrix improves its conductivity and enhances its response by capitalizing on the nature and properties of the nanoscale filler. In this direction, Zheng and coworkers investigated the microwave properties of the PVDF polymer with the nanofiller MnFe_2_O_4_/RGO.^58^ They found that the composites had a minimum reflection loss of 29.0 dB at 9.2 GHz at a 5 wt% loading of filler. Moreover, a high dielectric loss and magnetic loss occur due to synergistic effect between RGO and MnFe_2_O_4_, RGO and PVDF, and PVDF and MnFe_2_O_4_. This analysis shows that PVDF takes a part in impedance mismatching and improved the performance.

### 7.2 Thermosetting polymers

Versatile thermosetting-resin-based composites offer good adhesion, resistance to corrosion, high strength and stability. These polymers commonly establish a good dispersion and interfacial adhesion between the filler and the polymers. Epoxy resins are one of the most important thermosetting resins, especially for industrial applications. In general, polyurethanes (PU) and polyethylene terephthalate (PET) polymers (after epoxy) are used to suppress EMI pollution. However, these polymers do not respond in presence of EM waves due to their insulating behavior. Therefore, they are widely used with conducting polymers and carbon materials along with Fe materials. Moreover, thermosetting polymers like epoxy compounds are also used as binders with Fe materials that prevent the aggregation of Fe nanostructures and serve as ideal dispersing materials.

### 7.3 Elastomeric polymers

Elastomers are polymers which exhibit visco-elasticity and are bounded with weak intermolecular forces. These polymers are insulating in nature and have poor physico-mechanical properties (*e.g.* low Young’s modulus). Ethylene-vinyl acetate (EVA), ethylene-propylene-diene monomer (EPDM) and nitrile rubber (NBR) are some examples of these synthetic rubbers. Apart from some weaknesses, rubber has excellent weathering resistance, resistance to aging, and chemical resistance along with good compatibility with many kinds of fillers.^59^ Therefore, these elastomers have been used with magnetic Fe ingredients and conducting polymers or carbon materials, which improve its conductivity and enhance the EMI performance.

### 7.4 Other polymers

Apart from the polymer matrices discussed earlier, other polymers such as polyvinylpyrrolidone (PVP), polyvinyl chloride (PVC), poly(*p*-phenylenevinylene) (PPV), polypropylene (PP), polyvinyl butyral (PVB), polyvinyl alcohol (PVA), polyethylenimine (PEI) and polycarbonate, along with blends (PC (polycarbonate)/SAN [poly(styrene-*co*-acrylonitrile)]) and polymer composites have also been studied. Akinay *et al.*^60^ synthesized polyvinyl butyral (PVB)/Fe_2_O_4_ and (PVB)/NiFe_2_O_4_ composites and observed that the composites exhibit good RL_min_ performances in the 1–14 GHz range. In NiFe_2_O_4_/PVB composites, percolation of NiFe_2_O_4_ particles within the PVB matrix resulted in good RL_min_ values. In contrast, the overall microwave absorption performance was better in Fe_3_O_4_/PVB in comparison with (PVB)/NiFe_2_O_4_. In similar way, Yao *et al.*^61^ reported better EMI performance of PVC/graphene/Fe_3_O_4_ composites. PVC composites have negligible EMI SE_T_ due to their insulating behavior. In comparison to pure PVC, the addition of 5 wt% graphene and 5 wt% Fe_3_O_4_ nanoparticles form sufficient conducting interconnected graphene–Fe_3_O_4_ networks in the insulating PVC matrix. Hence graphene/Fe_3_O_4_/PVC obtained an improved *S*_T_ value compared to PVC/Fe_3_O_4_ and PVC/graphene composite.

## 8 Carbonaceous materials

**Fig. 8:**
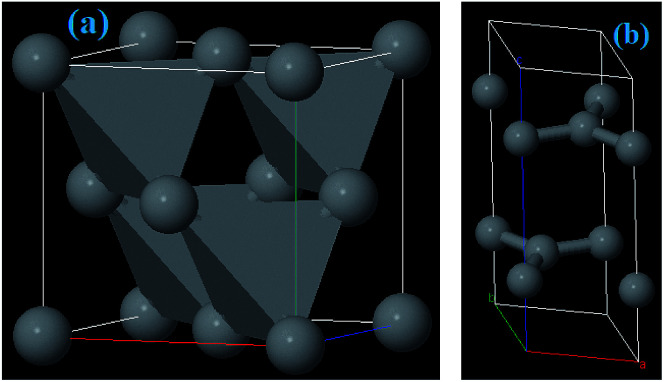
(a) Crystal structure of diamond and (b) graphite.

**Fig. 9:**
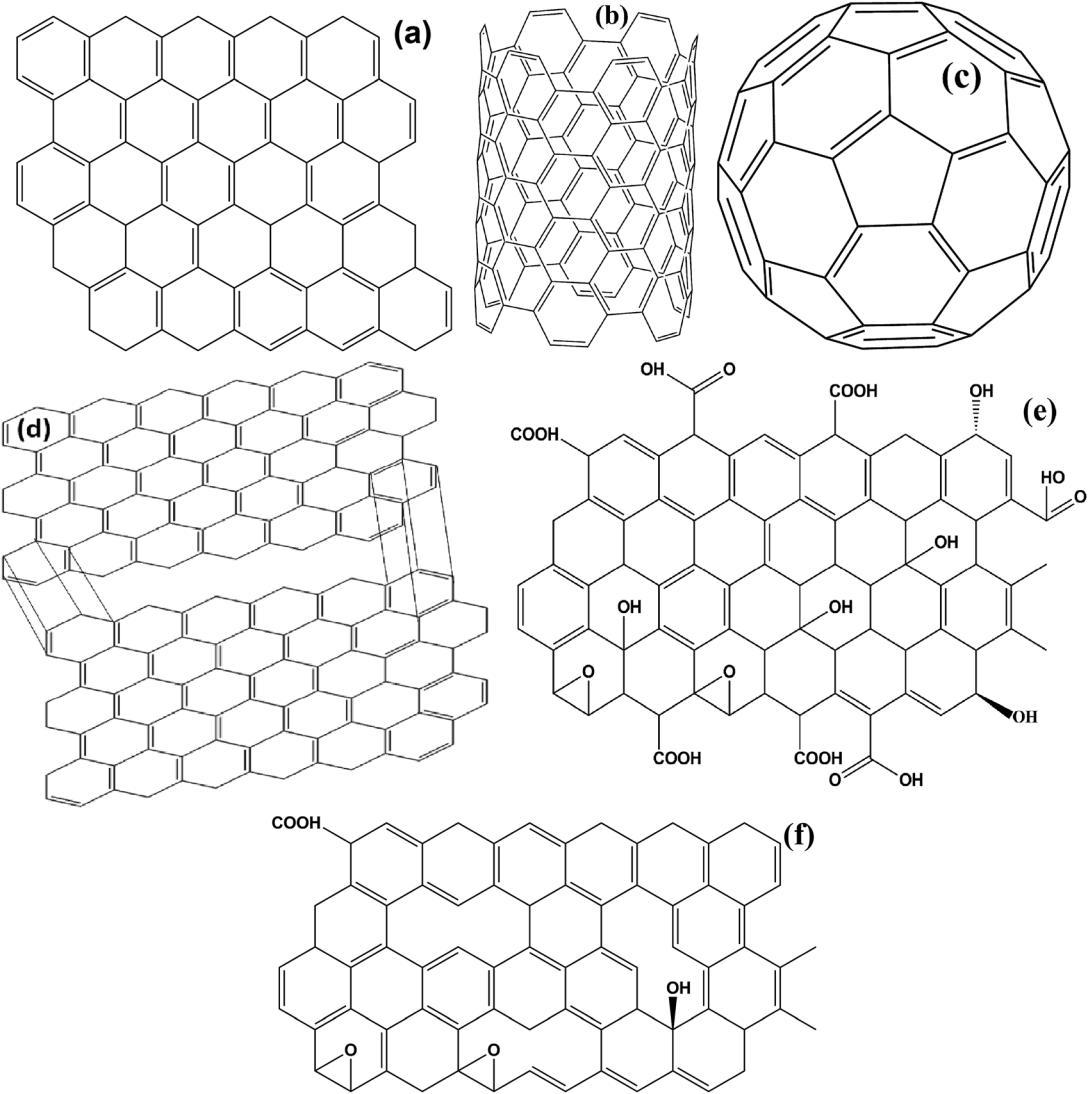
(a) 2D graphene, (b) carbon nanotube, (c) fullerene, (d) expanded graphite, (e) graphene oxide and (f) reduced graphene oxide.

Carbonaceous materials with unique characteristics such as low density, high permittivity, excellent conductivity, high chemical, thermal and mechanical stability are a current fields of growing interest scientifically as well as technically. These materials offer a great opportunity to fabricate a lot of varieties of new generic materials, with tunable optical, electrical, mechanical and magnetic properties. Most importantly, the high permittivity of carbonaceous materials establishes complementary behavior between the Fe ingredients and the carbon based materials that make it suitable for EMI applications. It is a well known fact that in the universe, after the evolution of hydrogen, helium, and oxygen, carbon (C) is the fourth most common chemical element. Pure carbon occurs in two main ordered lattice structures: diamond and graphite, shown in Fig. 8(a and b). Diamond has many industrial uses like cutting, and polishing of equipment, along with some scientific applications. Moreover, diamond is the hardest natural material, highly thermally conductive and electrically insulating (band gap ∼ 5.5 eV), as well as valuable and venerable; these properties cause it to be disfavoured in potential energy applications. On the other hand, graphite is soft, lubricating and electrically conductive. Furthermore, carbon possesses various allotropes, as depicted in Fig. 9(a–c), comprising 2D graphene, 0D buckminsterfullerene and 1D carbon nanotubes (single wall and multi wall). These lightweight carbonaceous materials and their derivatives with Fe ingredients serve as excellent candidates for the design of effective EM reflection/or absorption materials. A brief introduction to some carbon materials which are usually used in EMI shielding applications, along with their pro and cons, is given in a later subsection.

### 8.1 Graphite/expanded graphite

Graphite is a traditional carbon material which has a layered lattice consisting of hexagonal rings of carbon atoms attached by weak van der Waals forces in different planes. Within a plane, the carbon atoms are joined together by covalent bonds. As a low cost, lightweight lubricant graphite possess good electrical conductivity, a high aspect ratio, and good mechanical and thermal stability that establish it as attractive filler in several potential applications in the fields of electronic, optical and energy devices. However, the major drawback of graphite is its poor dispersion in solvents. Therefore, functionalized graphite, produced by HCl and H_2_SO_4_ acid treatment, is mostly used to prepare the composites.^62^ Apart from conventional graphite, more and more interest is being extended to expanded graphite. Expanded graphite (EG), obtained by thermal treatment, has many advantages, *e.g.*, EG is 2-dimensional, consisting of a small stack of graphite layers, low cost and has poor resistivity and high mechanical stability (Fig. 9d). The major problem of using these materials is their poor magnetic properties that restrict their practical application. Therefore, anchoring of Fe ingredients with graphite or expanded graphite integrates their magnetic properties due to the synergistic effect between iron and the graphite and thus enhancing the EMI performance in the microwave region.

### 8.2 Graphene

Graphene (G or GNS) is defined as a 2-dimensional (2D) allotrope of carbon atom formed by a single atomic layer of a honeycomb hexagonal lattice that hybridizes by sp^2^ bonding, as depicted in Fig. 9a. Graphene has an amazing mechanical strength with good elasticity, excellent electrical conductivity, superior thermal conductivity, extremely high surface area (∼2630 m^2^ g^−1^ theoretical value) and extraordinary electrical and thermal stability. Moreover, the theoretical dielectric loss of graphene is found to be superior than conventional oxide materials like ZnO, TiO_2_ or SnO_2_.^5^ Several preparation methods of graphene, including top-down or bottom-up approaches and chemical vapor deposition (CVD) have been reported till now. However, these physical methods do not offer the large scale production of graphene. Additionally, the lack of surface functionalities and the excessively high carrier mobility of graphene is also harmful for EM absorption, creating impedance mismatching between air and the material. Hence graphene’s derivatives such as graphene oxide (GO) and reduced graphene oxide (RGO) are more broadly used as alternative to graphene in practical applications.

#### 8.2.1 Graphene oxide (GO)

It has been demonstrated that when graphite is oxidized with strong oxidizing agents, the resulting attached oxygen functionalities, carboxyl, carbonyl, hydroxyl and epoxy groups (*i.e.* COOH, O–H) expand the layer separation within graphite along the *c* axis and make it hydrophilic. This hydrophilicity enables us to extract graphene oxide after water sonication (Fig. 9e). The most appealing property of GO is easy dispersion in either kind of solvent (organic or inorganic), because organic groups pave the way for GO to be modified easily by other materials. Moreover, GO can be well dispersed in a polymer matrix because of the strong and specific interactions (*e.g.* hydrogen bond) among the organic groups on the GO surface and the polymers. As reported by Samadi *et al.*^63^ for Fe_3_O_4_–GO/PVDF composites show better electromagnetic microwave absorption than pure PVDF. GO in Fe_3_O_4_–GO/PVDF composites does not only affect the reflection loss and absorption bandwidth but also has a great impact on the α-to-β phase transformation of the PVDF crystals. To evaluate quantitatively the EMI performance by GO we shall discuss its electro-magnetic properties. The disruption of sp^2^ bonding in GO diminishes its electrical properties. Hence GO acts as an electrical insulator, directly this is not very useful. However, the Fe components improve its conductivity to a certain extent. Furthermore, to recover the honeycomb structure of GO, different methods like reflux, hydrothermal and sol–gel approaches have been employed.

#### 8.2.2 Reduced graphene oxide (RGO)

**Fig. 10:**
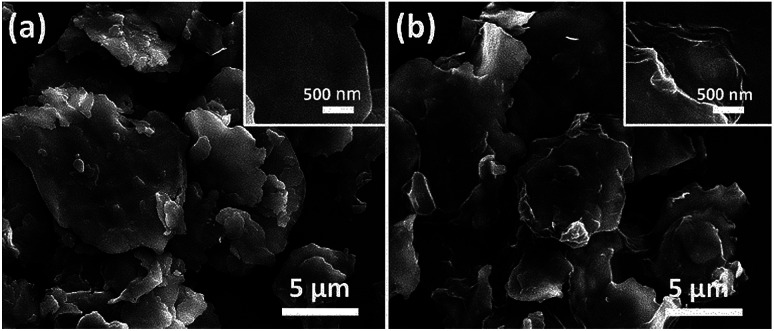
(a) SEM images of FCI and (b) RGO-coated FCI^66^ – reproduced by permission of the Royal Society of Chemistry.

**Fig. 11:**
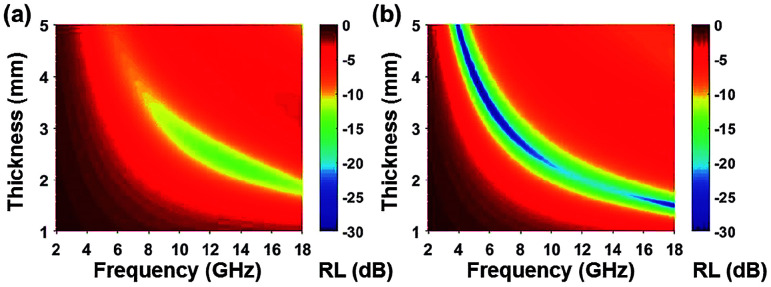
(a) Reflection loss mapping of FCI and (b) RGO-coated FCI with absorbers thickness from 1 mm to 5 mm in the frequency range of 2.0–18.0 GHz^66^ – reproduced by permission of The Royal Society of Chemistry.

**Fig. 12:**
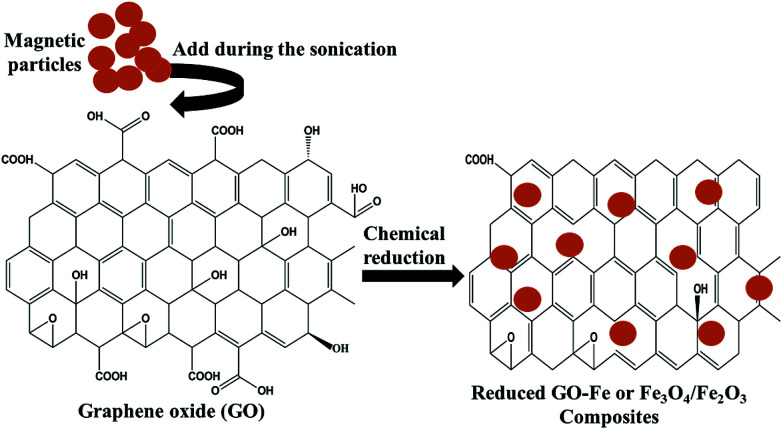
Chemical reduction method of preparation for Fe and RGO based composites.

Among all the carbon-derived materials, reduced graphene oxide (RGO) is a most promising material with diverse applications in several branches of science. In RGO, the oxygen functional group is removed using a reducing agent such as hydrazine hydrate, NaBH_4_ or NaOH *etc.* Reduced graphene oxide (RGO) is the most studied carbon derivative due to its cost effective preparation, good flexibility, superior electric/thermal conductivity and attractive barrier properties.^64^ Moreover, RGO comprises remanent functional groups and defects within the sheet which improve impedance mismatch, defect polarization relaxation and electronic dipole relaxation (Fig. 9f). All these groups and defects increase absorption rather than reflection, as can be seen in graphite and carbon nanotubes. For example, Wang *et al.*^65^ investigated the microwave absorption properties of chemically reduced graphene oxide. They observed that residual defects and organic groups within RGO not only improved the individual impedance matching but also produced energy transitions from the continuous states to the Fermi level. Furthermore, these peculiarities introduce relaxation polarization, defect polarization relaxation and electronic dipole relaxation which favor EM wave penetration and absorption. Compared with graphite and carbon nanotubes, reduced graphene oxide has a higher dielectric/magnetic loss by means of microwave absorption. Thus, due to the unique properties of RGO and Fe-based materials, as well as the synergistic effect between them, many reduced graphene oxide/Fe based composites for EMI shielding have been investigated. He and coworkers^66^ mixed reduced graphene oxide (RGO) nanosheets with the flaky carbonyl iron (FCI) as depicted in Fig. 10(a and b). They observed that FCI/RGO composites (−65.4 dB at 5.2 GHz at thickness 3.87 mm) lead to better microwave absorption properties compared with pure FCI (−13.8 dB at 13.7 GHz at thickness of 2.28 mm), as shown in Fig. 11(a and b). More interestingly, they used the delta-function method to see the contribution of typical dielectric dispersion behavior in FCI/RGO. It is anticipated that a smaller delta value gives better impedance matching. Since FCI/RGO possesses a larger area close to zero, which can directly explain the better matching of the characteristic impedance in FCI/RGO composites. Therefore, recent investigations have mainly concentrated on RGO and Fe-, Fe_3_O_4_- and Fe_2_O_3_-based composites due to their ease of preparation. In most of these cases the chemical reduction method is employed to fabricate the Fe- and RGO-based composites. In this method, Fe_3_O_4_ nanoparticles and GO are ultrasonicated/stirred followed by the addition of a reducing agent/surfactant and heat treatment by re-fluxing or hydro-thermal means, *etc.* which reduced the GO into RGO, as shown in Fig. 12.

### 8.3 Carbon nano tubes (CNTs)

Carbon nanotubes are unique one-dimensional (1D) nanostructures that can be understood hypothetically as a 1D quantum wire. These nanotubes belong to the fullerene family. Structurally, CNTs are a long, hollow structure with cylindrical walls framed by a honeycomb lattice (similar to graphene). Carbon nanotubes have received much recognition due to their intriguing electronic, mechanical (tensile strength is >60 GPa) and thermal properties.^67,68^ CNTs may be semiconductors or metallic depending on their structure and diameter. Furthermore, the high aspect ratio, low mass density (∼1.6 g cm^−3^), and wall integrity of CNTs enable them to serve them as superb nanofillers for improving the properties of composites.^22,62^ There are two main types of carbon nanotubes: single walled carbon nanotubes (SWCNTs) and multi-walled carbon nanotubes (MWCNTs).

#### 8.3.1 Single walled carbon nanotubes (SWCNTs)

Single walled carbon nanotubes (SWCNTs) are an allotrope of sp^2^ hybridized carbon atom, similar to fullerenes. A single sheet of carbon comprises the wall thickness all around the circumference (diameter ∼ 1.4 nm). The structure of SWCNTs is a cylindrical tube including six-membered carbon rings similar to graphite. Single walled nanotubes are a crucial type of carbon nanotube owing to their good electric properties compared to MWCNTs. The electrical properties of SWCNTs are distinctly different from their larger diameter MWCNTs counterparts due to their smaller diameters and larger aspect ratios. Because of this, the EM-absorbing properties of MWCNTs and SWCNTs are expected to be altogether different.^69,70^ The main flaws of SWCNTs are the complicated synthesis procedure, extremely high conductivity and poor magnetic properties. These characteristics of SWCNTs inhibit their use as excellent microwave absorbing materials. Although the incorporation of Fe materials improves their magnetic and electrical properties, as studied by Kuchi *et al.* in Fe_3_O_4_/SWCNT composites,^71^ SWCNTs still have been the subject of rather few studies.

#### 8.3.2 Multi walled carbon nanotubes (MWCNTs)

Multiwalled carbon nanotubes (MWCNTs) are one of the most preferable CNTs. Structurally, MWCNTs possess multiple layers of graphite superimposed and rolled in on themselves to make a tube shape. Moreover, these can be considered as a collection of concentric SWCNTs consisting of different diameters, lengths and natures. The distance between each layer is well known to be approximately 0.34 nm.^72^ MWCNTs are most promising 1D materials due to their attractive properties. Note that structural disorders, appearing in pristine MWCNTs during their development, are responsible for the unusual electrical and optical *etc.* properties of MWCNTs. These structural disorders might be Stone–Wales defects, atomic defects or in the form of vacancies and incomplete bonding defects *etc.* As the result of their high aspect ratio, large surface area and low percolation threshold, MWCNTs are favored as effective fillers rather than SWCNTs in terms of EMI shielding potential; despite this, their comparatively high cost limits their application to some extent.

The available literature on CNTs demonstrates that pure CNTs manifest low absorption but a significantly larger skin depth. However, the addition of Fe species to CNTs greatly improves their microwave absorption, as predicted by Che *et al.* and Qi *et al.* in the case of CNTs/CoFe_2_O_4_ and Fe/CNTs composites, respectively. The combination of Fe compounds with CNTs (*e.g.* Fe/CNTs or CNTs/CoFe_2_O_4_) presents better matching between the dielectric and magnetic losses. Moreover, observations have revealed that a fine dispersion of CoFe_2_O_4_ nanoparticles within the CNT matrix weakened the congregation of the CoFe_2_O_4_ particles, resulting in dipolar interaction and the resonance absorption effect, owing to the shape anisotropy.^3,73^

#### 8.3.3 Carbon fibers (CFs)

Similar to other carbon materials, carbon fibers (CFs) also possess a high mechanical strength modulus and a low density but a poor thermal expansion coefficient. CFs composed of fibers between (50 to 10 μm in diameter) mainly consisted of carbon atoms. Nevertheless, their lower magnetism and high conductivity increases impedance mismatching in EMI due to increasing their skin depth, similar to CNTs. Hence, modification of CFs with Fe, Fe_3_O_4_, Fe_2_O_3_ or alloys could be a useful approach to handle the above problem. Still, the high cost of CFs limits their potential for extensive use as effective fillers.

Apart from these fillers, graphitic carbon, carbon black and carbon coils have also been investigated for EMI applications. Although the large surface area of these fillers improves many properties, the major impedance to using these materials as fillers is the requirement for a high weight % ratio, which deteriorates the mechanical properties in case of these polymers.

## 9 Strategies for the preparation of effective EMI shielding materials

**Fig. 13:**
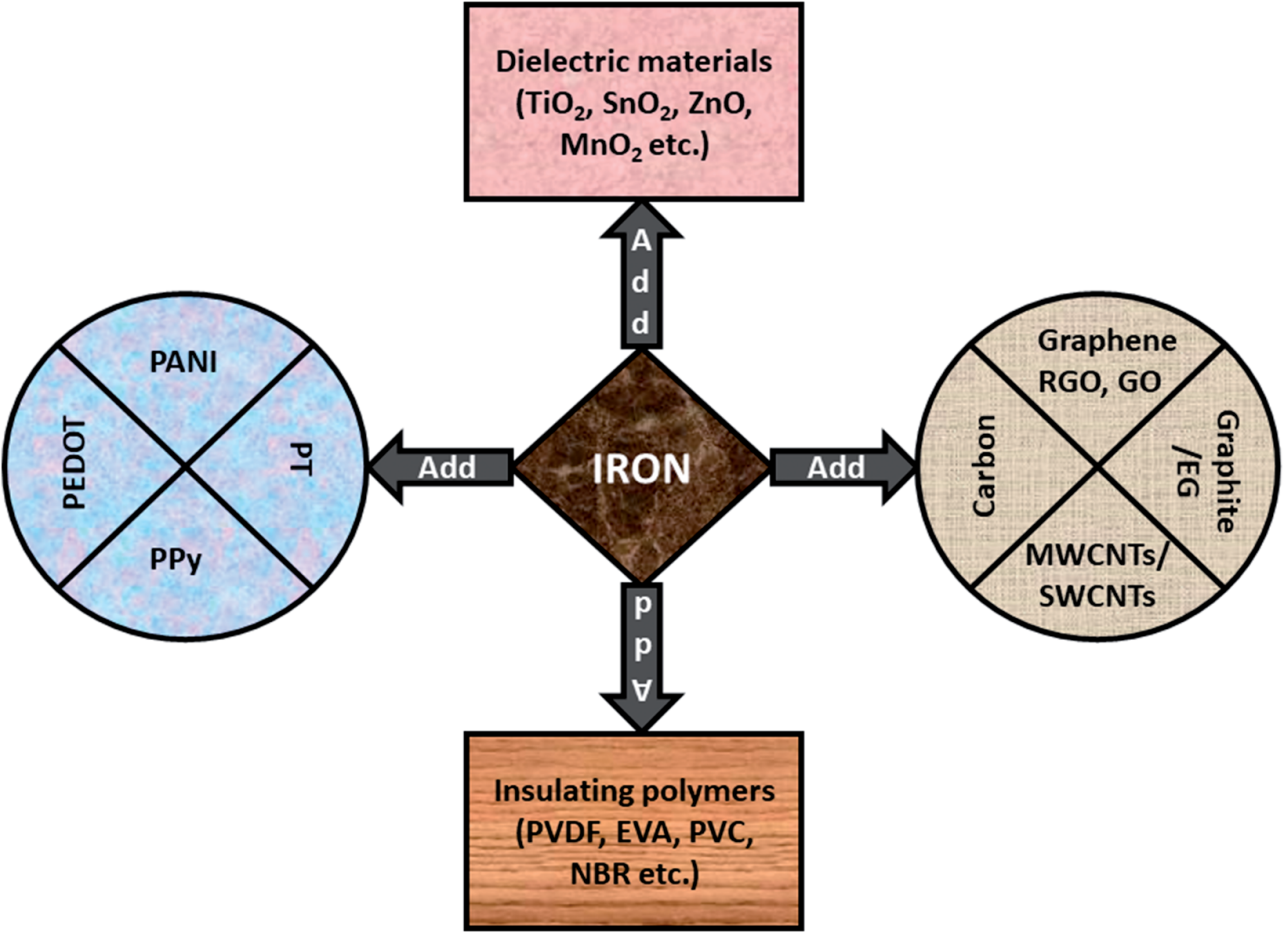
Iron can be used with carbonaceous materials, conducting polymers, dielectric materials or insulating polymers.

As we discussed in earlier sections, Fe-related materials offer significant improvements of the complex permeability *μ* which lead to a larger impedance matching value. Subsequently, these magnetic composites shows strong interface polarization (in case of multi-interface materials) which offer advantages with respect to conversion of the incidence EM thermal energy into thermal energy. Keeping this in mind, different strategies for EMI shielding materials and magnetic absorbers have been proposed by scientists, as shown in Fig. 13, and explained in later subsections.

### 9.1 Hierarchical/porous structure or divalent/trivalent ion substitution in ferrites

The first strategy is to make a hierarchical structure of Fe-based materials that improve the performance. It is well established that there are two factors that mainly affect microwave absorption: dielectric loss and magnetic loss. Permittivity and permeability result from electronic polarization, ion polarization, dipole polarization, natural resonance, exchange resonance, hysteresis and eddy losses, in which size, distinct geometrical morphology and crystal structure may have an essential impact. Therefore, several scientists have merely paid attention to complicated morphologies of Fe materials. To my knowledge, Fe-based structures including flakes of α-Fe_2_O_3_ and Fe,^41,74^ octahedral Fe_3_O_4_,^75^ Fe nanowires, urchin-like structures of Fe_3_O_4_, α-Fe_2_O_3_,^76^ nanocapsules such as α-Fe/ZnO, α-Fe/SnO and Fe/Fe_3_B/Y_2_O_3_,^77^ dendritic structures of Fe, Fe_3_O_4_ and α-Fe_2_O_3_,^40^ and loose nano-Fe_3_O_4_ (ref. 78) have until now been exposed as efficient EMI shielding materials. Shang *et al.*^75^ have shown that, in the case of octahedral Fe_3_O_4_ nanoparticles, their anisotropic structure has excellent magnetic characteristics on account of its shape anisotropy. Moreover, the octahedral structure is also advantageous for reflection scattering from multi edges. Zhang *et al.*^79^ made a comparative study of sphere-shaped particles and flake-shaped carbonyl iron particles. They found an optimal reflection loss (12.2 dB at 4.4 GHz at 1 mm thickness) in flake carbonyl iron particles, which was better than that in sphere-shaped particles. Similarly, porous Fe materials have also been widely studied due to their large specific surface area, pore volume and attractive magnetic properties. In addition, the porous structure endows multiple reflection properties to the material which contribute to its total effectiveness. Li *et al.*^80^ noticed improved absorption in Mn_*x*_Fe_3−*x*_O_4_ hollow/porous spherical chains. These findings in Mn_*x*_Fe_3−*x*_O_4_ occurred due to the porous and hollow structures, the oriented arrangement of the nanocrystals and Mn^2+^ substitution, because Mn^2+^ replacement induced a dual-frequency absorption in Mn_*x*_Fe_3−*x*_O_4_ in the 2–18 GHz range. Since substitution has great impact on the EMI properties, divalent and trivalent impurities such as Bi, Al, Nb, Cu, Ni, Zn are therefore widely substituted in several ferrites, as listed in Table 1 where 
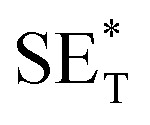
 is the total shielding effectiveness and RL is the reflection loss. Moreover, the line width of magnetic resonance is directly related to the magnetocrystalline anisotropy of the ferrite system. To achieve broad-band and strong magnetic loss in ferrites, a high anisotropy field (*H*_a_) is needed. In this context, doping of divalent or trivalent elements in ferrites enhances their EMI performance. For instance, Song *et al.*^81^ studied the influence of Al-substitution on the microwave absorbing properties of Ba_1.5_Sr_0.5_CoZnA_1−*x*_Fe_12−*x*_O_22_ (for *x* = 0–1) hexaferrites in which domain wall resonance and natural resonance were found to responsible for effective microwave absorption. Zhang and coworkers^82^ observed the effect of NdCo substitution of SrFe_12_O_19_ ferrites, *i.e.* Sr_1−*x*_Nd_*x*_Fe_12−*x*_Co_*x*_O_19_ (*x* = 0–0.4). The complex permittivity of these ferrites resulted from the significant contributions of Nd^3+^ and Co^2+^ ions. Further dielectric properties of SrFe_12_O_19_ ferrites arise mainly because of the interfacial polarization and intrinsic electric dipole polarization which occurs as a result of electron hopping between ions of different valence states. Thus, Nd^3+^ ions preferentially substitute for Sr^3+^ ions, and Co^2+^ ions preferentially substitute for Fe^3+^ ions, which enhances the electron hopping. In the meantime, magnetic loss in ferrites usually originates from natural ferromagnetic resonance and domain wall resonance, but domain wall resonance was found in the low-frequency region (<2 GHz). On the other hand, resonance due to the spin rotational component occurs at high frequency regions. Thus, the magnetic loss in the M-type strontium hexaferrite/paraffin was found to be due to natural resonance. Li *et al.*^83^ have studied Fe_3_O_4_/NiFe_2_O_4_/Ni heterostructured porous rods in which they observed that controlling the NiFe_2_O_4_ interface layers and Ni content can improve impedance matching and dielectric losses, thereby leading to lighter weight, a stronger absorption, and a broader absorption band of Fe_2_O_4_/NiFe_2_O_4_/Ni compared to Fe_2_O_4_.

**Table 1:** Fe based microwave absorber

Fe based components					
Materials	Methods	Thickness/mm	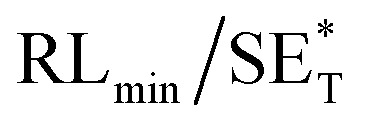 /dB	Frequency/GHz	Ref.
Porous Fe particles	Corrosion technique	1.8	−42.2	13.2	84
Porous CI flake	Two-step approach	3.5	−41.8	4	85
α-Fe_2_O_3_ nanoflake	Hydrothermal	5	−41.67	2.8	41
Loose nano-Fe_3_O_4_	Hydrothermal	5.5	−30.33	13.54	78
Hexagonal Fe flakes	Hydrothermal	1.1	−15.3	12.2–16.6	74
Fe nanoparticles	Ball milling	1.4	−23.67	15.24	75
Urchin like α-Fe_2_O_3_	Two step process	1–5	−9.2	3.76–8.15	76
Urchin like Fe_3_O_4_	Two step process	3–4	−29.96	3.76–8.15	76
Octahedral Fe_3_O_4_	*In situ* molten salt	1.4	−23.67	15.24	75
γ-Fe_2_O_3_ dendritic	Hydrothermal	4	−50	2–13	40
Fe_3_O_4_ dendritic	Hydrothermal	4	−53	2.2	40
Fe dendritic	Hydrothermal	3	−25	2.5	40
Fe_3_O_4_ nanosphere	Hydrothermal, calcination	3	−12	12.7	86
Porous flower like Fe_3_O_4_	Reflux	2	−28.31	13.2	87
Coin-like Fe	Reduction	1.4	−53.2	16	88
SiC–Fe_3_O_4_ nanowires	Polyol approach	—	−51	8.6	89
γ-Fe_2_O_3_ nanosphere	Hydrothermal, calcination	3	−18	14.8	86
Fe nanowire/epoxy	Chemical vapour deposition	2	−47	9.4	90
Porous α-Fe_2_O_3_ nanosphere	Hydrothermal, calcination	3.5	−25	13	86
Mn_*x*_Fe_3−*x*_O_4_ spherical chains (*x* = 0.74)	Solvothermal	2.6	−52.8	10	80
Coinlike α-Fe_2_O_3_@CoFe_2_O_4_	Solvothermal	2	−60	16.6	45
Co/CI nanoparticles	Electroless plating process	1.8	−27.8	10.4	91
Co_*x*_Fe_3−*x*_O_4_ (*x* = 0.9)	Solvothermal	2	−41.09	12.08	92
Fe/ZnFe_3_O_4_/CI	Ball milling, *in situ*	1.5	−47	6.2	93
Ni._5_Zn_0.5_/Fe_2_O_4_/Co flake	Co-precipitation	1.5	−33.8	11.5	94
Ni_1−*x*_Zn_*x*_/Fe_2_O_4_ (*x* = 0.5)	Sol–gel	5	−29.6	6.5	95
Ni_1−*x*_Co_*x*_/Fe_2_O_4_ (*x* = 0.3)	Co-precipitation	2	−18	2.45	96
Zn_*x*_Cu_1−*x*_/Fe_2_O_4_ (*x* = 0.8)	Sol–gel, auto-combustion	3	4–9	8.2–12.4	97
Cu_*x*_Ni_0.4−*x*_Zn_0.6_/Fe_2_O_4_ (*x* = 0.2)	Ceramic method	1	−60	0.01	98
α Fe/Fe_3_B/Y_2_O_3_	Melt-spun technique	4	−33	4.5	99
Ba_1−2*x*_La_*x*_Na_*x*_Fe_10_Co_0.5_TiMn_0.5_O_19_ (*x* = 0.1)	Solid state reaction	1.3	−45.94	8.33	100
Ba_1−*x*_Ce_*x*_Fe_12_O_19_ (*x* = 0.21)	Sol–gel	10	−20.47	16.22	101
Ag_3_PO_4_/SrFe_12_O_19_	Hydrothermal	5.1	−63.18	4.72	102
Sr_1−*x*_Nd_*x*_Fe_12−*x*_Co_*x*_O_19_ (*x* = 0.4)	Sol–gel, autocombustion	1.9	−22	16.2	82
BaFe_12−*x*_Ti_*x*_O_19_/CI (*x* = 0.2)	Sol–gel, physical blending	4.5	−30.7	5.67	103
Sr_1−*x*_Ba_3−*x*_Co_2_Fe_24_O_41_ (*x* = 1.5)	Sol–gel	5	−48	17.6	39
Ba_3_Co_2_Fe_24_O_41_	Sol–gel	5	−50	4.5	104
(CuZn)_*x*_Co_2(1−*x*)_Fe_24_O_41_ (*x* = 0.3)	Sol–gel	25	−29	11.4	105
Ba_2−*x*_Dy_*x*_Zn_2_Fe_28−*y*_Mn_*y*_O_46_	Sol–gel	—	−55	11.62	106
BaSrCo_2−*x*_Ni_*x*_Fe_12_O_22_ (*x* = 0.5)	Solid state reaction	1.2	>−45	12	107
BaSr_0.5_CoZnFe_12−*x*_Al_*x*_O_22_ (*x* = 0.3)	Sol–gel	3	−19	11.5	36
Sr(MnTi)_*x*_CoZnFe_12−2*x*_O_19_ (*x* = 1)	Aqueous combustion	2	−22.7	11.5	108
SrZnCoFe_16_O_17_	Combustion synthesis	2.6	−33.6	10.4	109
Sr_0.9_Nd_0.1_ZnCoFe_16_O_17_	Ceramic	4.6	−21	9.6	37
SrZn_2−*x*_Co_*x*/2_Ni_*x*/2_Fe_16_O_17_ (*x* = 0.4)	Co-precipitation	1.8	−29.11	14.57	38
Ba_0.5_Sr_0.5_Co_*x*_W_*x*_Fe_12−*x*_O_19_ (*x* = 0.2)	Ceramic	2.8	−15.2	11.22	110

### 9.2 Alloying of Fe with other metals

The second strategy is to make alloys of Fe with other materials. In this case the other material may be either magnetic, such as Co or Ni, or nonmagnetic like Zn, Cu *etc.* It is well established that ferrites have a low Curie temperature *T*_C_, moderate saturation magnetization (*M*_s_) value and a negative thermal coefficient of resistivity, while pure Fe suffers a stability problem in air along with forefront reflection due to its high conductivity. Nevertheless, a material with high saturation magnetization *M*_s_ is required for better microwave absorption/EMI performance. Therefore, Fe based alloys can be used as an ideal candidate because their combined properties not only improve *M*_s_ and the Curie temperature (*T*_C_) but also provide environmental stability to Fe. In this regard, intermingling of the properties of metals could be achieved by alloying two (binary alloy), three (ternary alloy) or more metallic elements. In EMI applications, alloying provides strong bonding between the alloy filler and the matrix and also increases the interfacial polarization, saturation magnetization and permittivity *etc.*, which cannot be obtained in pure metals. For example, a 20% Fe content in an FeNi alloy shows interesting physical properties in comparison with pure Fe or Ni, and also the FeNi alloy is cost-effective compared to magnetic materials such as Ni, Co and Fe_3_O_4_. Extensive research has been conducted on Fe alloying with Co, Ni, Cu, Si and Al elements within dielectric materials like ZnO, carbon (graphite, CNTs *etc.*), polymers such as PANI (conducting) and PVDF, and EPDM (non-conducting) matrices, respectively.^111–115^ These matrices enhance the permittivity of the alloy filler and make it low in weight and flexible. For example, Feng *et al.* have shown dual dielectric relaxation in the case of FeNi@C nanocomposites which occurs due to a cooperative consequence of the FeNi–C interfaces and dielectric carbon.^116^ Therefore, the synergy of dielectric and magnetic losses in FeNi@C provides excellent microwave absorption performance. Meanwhile, enhanced EMI shielding effectiveness (*S*_T_) was obtained by Choi *et al.* in FeCo hollow fibers mixed with ethylene propylene diene monomer (EPDM).^117^ In the FeCo/EPDM composites an increased number of conducting paths were formed because of the high aspect ratio of the fibers, which enhanced the reflection loss by impedance mismatching. Some of the studied alloy systems are listed in Table 2.

**Table 2:** Fe alloying based microwave absorber

Materials	Method	Thickness/mm	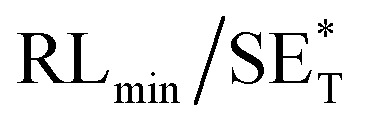 /dB	Frequency/GHz	Ref.
Fe_3_O_4_ coated Fe_0.65_Co_0.35_ flakes	Solvothermal	5.89	−41.4	2.02	118
Ni–Fe–P/PET	Electrodeposition	—	69.2–80.3*	1.5	119
FeCo/EPDM	Electroless plating process	—	30*	12.5	117
Fe–Si–Al/PP	Two roller mixer	—	−9.3	0.8	120
Ni–Fe–P/TS	Electroless plating process	—	60*	1.5	121
FeCo/ZnO	Hydrothermal	1.5	−31	5.5	122
FeNi/CFs	Two-step electrodeposition	2	42*	0.03–0.1	111
FeNi@C	Hydrothermal	2	−47.6	3.17	116
FeNi_3_@C@RGO	Three step reaction	2.6	−47.6	10.2	114
FeCo@SiO_2_@MWCNT	Two step reaction	3	−35	18	123
FeCo@SnO_2_@graphene@PANI	Three step reaction	3	−39.8	6.4	124
Co_3_Fe_7_@C yolk–shell	Hydrothermal, heat treatment	2	−35.3	9.1	125
FeCoNi-EG	Electroless plating process	3	−28.8	13.5	126
FeCo@SiO_2_@MWCNTs	Multi step processes	3	−35*	18	127
Carbon–FeCu@CNBs	Sol–gel, reduction	7.5	−21.02	12.21	128
FeCo_13_@C	Three steps	1.5	−6.7	11.1	129
FeCo/graphite nanoflakes	Jet milling, acid treatment	2	−30.6	7.4	130
Fe_*x*_Ni_1−*x*_@PVDF@MWCNT	Acid treatment, melt mixing	2.5	−58*	8–12	115
CI/Fe_91_Si_9_	Blending technique	3	−45	5.2	131

### 9.3 Core@shell structures

**Fig. 14:**
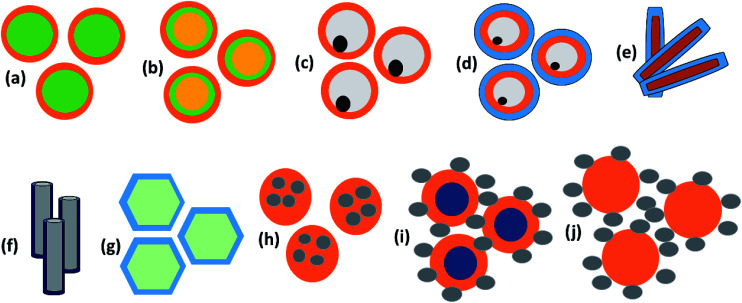
Schematic representation of the different types of core@shell structures.

The third strategy for achieving high-performing EMI materials is the fabrication of a core–shell structure of Fe-related materials, which is indicated by the term core@shell (core/shell or core–shell). Core@shell structures have emerged as a type of important nanostructure for various applications in different branches of sciences such as electronics, chemistry, biomedicine, energy, optics *etc.* Core@shell nanostructures could be attributed as a new kind of important functional material consisting of different functional compositions on the nanometer scale. The core and the shell can be made by two different materials such as organic/inorganic and *vice versa*, or by the same material, *i.e.* organic/organic or inorganic/inorganic, with distinct structures. Core and shell properties either arising from the core or the shell. These materials can be modified by varying the building materials or the core or shell thickness ratio. The core and shell occur in different forms, as shown in Fig. 14. The core may be a single sphere wrapped by single shell (Fig. 14a), or a multi-shell (Fig. 14b). Moreover, the shell might be hollow, in which a small sphere as the core is trapped in to shell. This type of core@shell is known as a yolk–shell structure (Fig. 14c). On other hand, a yolk–shell also can be multi-shell structure (Fig. 14d). In particular, the core@shell structure might take the form of rod-, tube- or hexagonal-flake-type morphologies (Fig. 14e–g). Furthermore, the core can be an accumulation of small spheres (Fig. 14h) within the shell. The core@shell structure might take the form of smaller spheres attached on the surface of the shell (Fig. 14i); instead of a continuous layer shell, it might also take the form of smaller spheres attached to a core sphere (Fig. 14j). The challenge in the preparation of core@shell nanoparticles (NPs) is to find a simple, cost-effective and less time-consuming strategy with minimum environmental impact. Du *et al.*^23^ prepared a 14a-type core@shell Fe_3_O_4_@C structure with 500 nm Fe_3_O_4_ microspheres. Observation revealed that carbon coating on the Fe_3_O_4_ microspheres increased the complex permittivity, and improved impedance matching occurred due to multiple relaxation processes. On the specific thickness of shells, Fe_3_O_4_@C showed an unusual dielectric behavior that favored a strong reflection loss, even at high frequencies. On other hand, Guo *et al.*^132^ formed a mesoporous α-iron oxide@*n*SiO_2_@*m*SiO_2_ multi-layer 14b-type core–shell structure. The α-iron oxide@*n*SiO_2_@*m*SiO_2_ composites showed improved electromagnetic interference (EMI) shielding effectiveness (SE) compared to the pure hematite materials. Yu *et al.* produced a yolk–shell Fe_3_O_4_@ZrO_2_ core@shell structure of the 14c type.^133^ They analyzed the effect of temperature on reflection loss and observed that even at 500 °C, Fe_3_O_4_@ZrO_2_ sustained over 90% of its reflection loss (RL) value with respect to its room temperature properties. Liu and co-workers present^134^ a 14d-type yolk core–shell. The unique morphology, well-defined shells, favorable magnetization, large specific surface area, and high porosity of these double-shelled Fe_3_O_4_@SnO_2_ yolk–shell microspheres significantly enhanced their microwave absorption characteristics. The exceptional microwave absorption properties of these Fe_3_O_4_@SnO_2_ yolk–shell microspheres may be ascribed to the distinctive double-shelled yolk–shell structure and the synergistic effect between the magnetic Fe_3_O_4_ cores and the dielectric SnO_2_ shells. Chen and coworkers generated Fe_3_O_4_@carbon nanorod (14e-type) structure *via* a three-step process.^135^ The outstanding EM wave absorption properties of these porous Fe_3_O_4_@carbon core/shell nanorods were ascribed to complementary behaviour between the dielectric loss and the magnetic loss as well as the unique structure of the porous Fe_3_O_4_@carbon structure. Furthermore, the Fe_3_O_4_@TiO_2_ core/shell nanotubes type of core@shell (14f-type) morphology was predicted by Zhu and coworkers,^136^ in which the eddy current effect decreased effectively and an improvement in anisotropy energy was observed due to the presence of the TiO_2_ shells. The maximum reflection loss was obtained −20.6 dB at 17.28 GHz with a 5 mm thickness in the tube like core@shell structure. Liu *et al.* have shown^137^ a 14g-type core@shell structure of Fe@SiO_2_ microflakes which demonstrated excellent microwave absorption performance of compared to Fe microflakes. In this case, effective balancing between the dielectric loss and magnetic loss improved the impedance matching. The structure of the Fe@MoS_2_ composite shows a 14h type core@shell structure, as explained by^138^ Pan *et al.*, in which 2 dimensional MoS_2_ nanosheets were distributed on porous coin-like Fe micro-sheets. The addition of MoS_2_ with Fe controls the permittivity and improves the impedance matching. Therefore, an optimal reflection loss was obtained around −37.02 dB at a coating thickness of 2.0 mm. In Fe@MoS_2_ composites, the magnetic loss is dominant over the dielectric loss. Among all magnetic losses, such as hysteresis loss, domain wall resonance loss, natural ferromagnetic resonance loss and eddy current loss, natural ferromagnetic resonance loss and the eddy current effect normally play a vital role in Fe@MoS_2_. Therefore, efficient microwave absorption can be attributed to improved impedance matching and the synergistic effect between the magnetic loss and the dielectric loss. Liu *et al.* presented excellent microwave properties with 14i-type iron oxide cores and hierarchical copper silicate shells.^139^ A maximum reflection loss value of these Fe_3_O_4_@Cu-silicate microspheres of 23.5 dB was achieved at 7 GHz with a thickness of 2 mm. The high porosity, large specific surface area and synergistic effect of both the magnetic Fe_3_O_4_ cores and the hierarchical copper silicate shells and their unique morphology plays an important role for impedance matching in microwave absorption applications. Zhu *et al.*^140^ produced the 14j-type structure in a Fe_3_O_4_ polyelectrolyte (PE)@PANI/paraffin composite. The Fe_3_O_4_-PE@PANI/paraffin composite exhibits an RL_min_ of −6.5 dB at 14.3 GHz. In the above nanocomposites, the peculiar structural interfaces produce interfacial relaxation between the Fe_3_O_4_ nanoparticles and PANI hollow spheres, which is also beneficial for microwave absorption.

In brief, Fe-based core–shell composites are of great interest due to their potential applications, because the core–shell structure have several advantages such as the confinement effect, interfacial polarization, complementary behavior, and core-corrosion protection. Therefore, a variety of core@shell composites with satisfactory EM wave absorption have been studied till now. These structures enhance microwave absorption due to their specific structure, *e.g.* a hollow multi-shell favors multiple reflections; meanwhile their porous nature and multiple interfaces increase interfacial polarization. Accordingly, the core structure might be binary (containing two different elements), ternary (containing two different elements) or quaternary core@shell (containing four different elements).

#### 9.3.1 Binary composites

Different strategies have been employed to fabricate binary core@shell structures for microwave applications. The binary core@shell can be prepared in the following ways:

##### 9.3.1.1 Magnetic core@magnetic shell

In this case, the core and shell are both prepared by magnetic materials. The core can be any magnetic element, alloy, ferrite or ceramic. Wang *et al.*^141^ studied the same type of Fe_3_O_4_/Co core@shell type structure. In this work, they showed that Co nanoshells form surface layers over the Fe_3_O_4_ core which greatly enhance the conductivity and permittivity. Moreover, free electrons in a metallic Co nanoshell can move freely within it. These charge carriers accumulate at the Fe_3_O_4_/Co interface, and form a structure similar to a boundary-layer capacitor and generate interfacial electric dipolar polarization. The permeability could be explained by hysteresis loss, domain-wall resonance, the eddy current effect and natural resonance. They excluded hysteresis loss because the applied microwave field was weak. Domain-wall resonance usually takes place at a frequency lower than the gigahertz range. Furthermore, they observed a high skin depth with respect to the diameter of the materials’ grain size, hence the eddy current can be precluded. For these reasons, natural resonance is deemed to be the main magnetic loss mechanism for the Fe_3_O_4_/Co sample. Despite this, the magnetic core@magnetic shell shows superior EMI performance, but suffers from a dual magnetic loss which decreases the dielectric loss and support to impedance mismatching.

##### 9.3.1.2 Magnetic core@dielectric shell

**Fig. 15:**
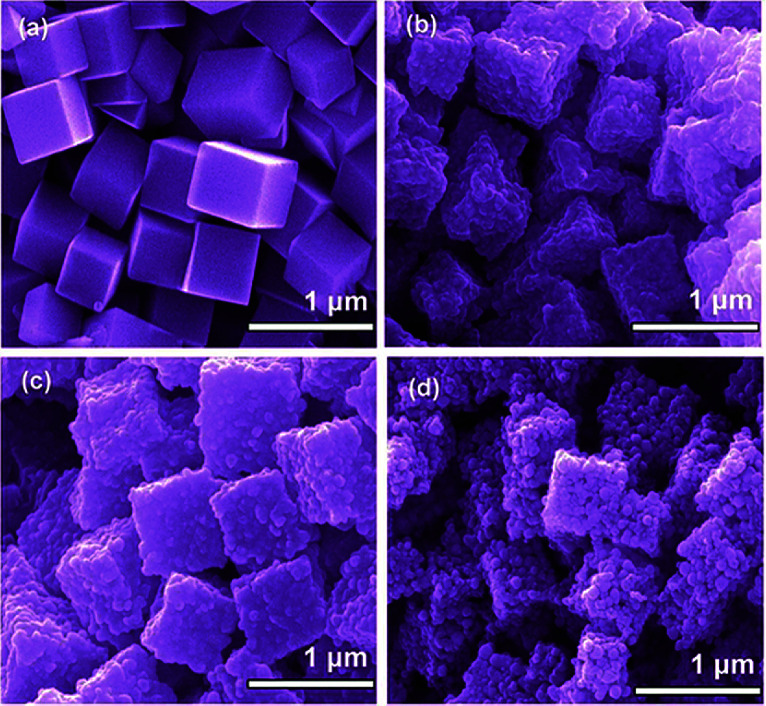
SEM images of the as-prepared PB nanocubes (a) and Fe/C nanocubes obtained at different pyrolysis temperatures: (b) 600 °C, (c) 650 °C and (d) 700 °C  ^150^ – reproduced by permission of the Royal Society of Chemistry.

**Fig. 16:**
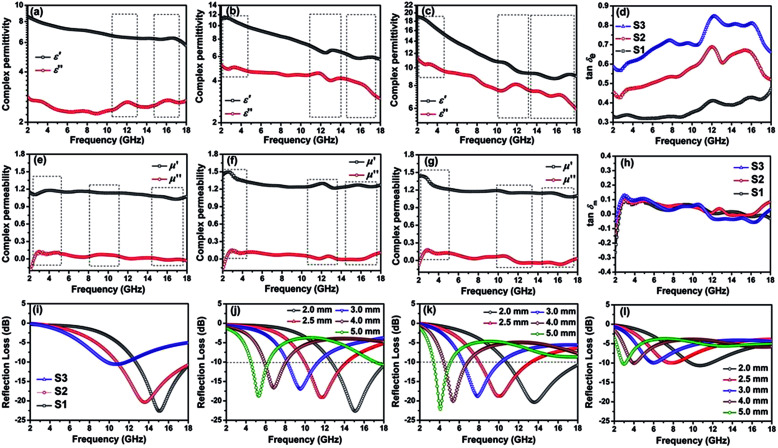
Complex permittivity of S_1_ (a), S_2_ (b), S_3_ (c), and their dielectric loss tangents (d); complex permeability of S_1_ (e), S_2_ (f), S_3_ (g), and their magnetic loss tangents (h); microwave reflection losses (absorber thickness = 2 mm) of Fe/C nanocubes (i), and reflection losses of S_1_ (j), S_2_ (k), S_3_ (l) with variable absorber thicknesses here S_1_, S_2_, and S_3_ indicate that their final temperature was set to 600 °C, 650 °C, and 700 °C, respectively^150^ – reproduced by permission of the Royal Society of Chemistry.

In this case the core is selected as magnetic and the shell is dielectric. The core may be any magnetic element, alloy, ferrite or ceramic, similar to those detailed in magnetic core@magnetic shell section. As a dielectric source, several dielectric materials such as Al_2_O_3_, TiO_2_, ZnO, ZrO_2_, SnO_2_, carbon materials and polymers have been investigated so far, as shown in Table 3. Among all core@shell structures, the magnetic core@dielectric shell is considered to be a highly desirable core–shell structure, because protective encapsulation on the surface of core prevents oxidation and agglomeration of the Fe metal nanoparticles. Additionally, the above type of core@shell material works as bridge between dielectric and magnetic losses. Hitherto, a lot of studies have been performed on Fe-based core@shell nanostructures. For example, Fe_3_O_4_@ZnO core–shell,^142^ ZnO-coated iron nanocapsules,^143^ SnO-coated-Fe(Sn) nanocapsules,^77^ Fe@Al_2_O_3_,^144^ Fe_3_O_4_@SnO_2_ double shells,^134^ Fe@SiO_2_ nanoflakes,^145^ spinel Fe_3_O_4_@ TiO_2_,^146^ Fe nanoparticles with amorphous Al_2_O_3_/FeO_*x*_ composites shells,^147^ and yolk–shell Fe_3_O_4_@ZrO_2_ (ref. 133) have been investigated as effective microwave absorbing materials. Liu *et al.*^148^ reported the synergistic effect of magnetic loss and dielectric loss in the Fe/SnO_2_ nanocapsules which enhanced its microwave absorption properties. They found two typical dielectric resonances at 3.8 and 16 GHz arising due to a synergistic effect of the Fe nanoparticles cores and SnO_2_ shells. An additional peak at 14 GHz could be explained as spin wave excitation. In the intervening time, they observed that the *μ* values indicate a peak at 3.2 GHz due to natural resonance within the Fe/SnO_2_ nanocapsules. They explained that the natural resonance might be the result of a surface/small size effect and spin wave excitations of the Fe/SnO_2_ nanocapsules.^149^ Similarly, Qiang *et al.*^150^ prepared Fe/C nanocubes obtained from Prussian blue (PB) nanocubes at varying pyrolysis temperatures (ranging from 600–700 °C) for their EMI properties. Fig. 15(a–d) shows SEM images of the Prussian blue (PB) nanocubes and Fe/C at different pyrolysis temperatures of 600 °C, 650 °C, and 700 °C. Fig. 15a shows the PB nanocubes with smooth surface and an edge length of around 600 nm. At high temperature pyrolysis, the PB nanocubes are *in situ* converted into Fe/C composites (Fig. 15b and c) but at 700 °C a little shrinkage in the cubic skeletal structure was observed. Fig. 16(a–c and e–g) shows the complex permittivity and permeability of Fe/C nanocubes in the frequency range 2–18 GHz. It is observed that the three samples exhibit different complex permittivity and permeability at different pyrolysis temperatures. Generally, the values of *ε*′/*μ*′ and *ε*′′/*μ*′′ are related to the electrical conductivity and magnetic losses of the EM absorbers. A high electrical conductivity is advantageous for a high complex permittivity while magnetic loss mainly comes from hysteresis, domain wall resonance, natural ferromagnetic resonance, and the eddy current effect. It is well established that both the carbon material and iron component are electrically conductive; the electrical conductivity of the carbon materials is more sensitive to the pyrolysis temperature with respect to the metal iron. Thus the improved graphitization degree of carbon components in Fe/C nanocubes increase the complex permittivity while the natural ferromagnetic resonance is the primary reason for the magnetic loss. Fig. 16(d and h) represent the dielectric/magnetic loss tangents, which indicate that dielectric loss ability is regularly improved with increasing pyrolysis temperature, whereas magnetic loss tangents varies in the limited range. It is clear from Fig. 16(f–l) that different Fe/C nanocubes display different EM absorption responses. The optimum RL was obtained at 650 °C, due to its promising dielectric loss and magnetic loss, as well as improved matched characteristic impedance.

**Table 3:** Binary Fe based microwave absorber

Materials	Methods	Thickness/mm	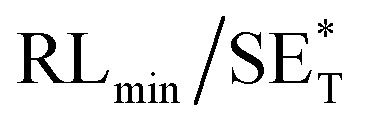 /dB	Frequency/GHz	Ref.
**Binary composites**					
Fe_3_O_4_@TiO_2_ nanotubes	Three-step process	5	20.6	17.28	136
Fe_3_O_4_@C yolk–shell	Chemical reduction	3	−45.8	10.6	152
Fe_3_O_4_ sphere@C	*In situ* polymerization	2	−20.6	13.4	23
Fe@NiFe_2_O_4_	Ball milling	1.5	−27	11.2–18	153
Fe@ZnFe_2_O_4_	Ball milling	1.5	−39	11.2–18	153
Fe_3_O_4_@ZrO_2_	Sol–gel	2	22	6	133
Fe_3_O_4_@MnO_2_	Hydrothermal	3	−43.6	9.2	154
Fe_3_O_4_ nanocrystal@ZnO	Heterogeneous nucleation	3.5	−22.69	10.08–15.97	155
Porous Fe_3_O_4_/carbon nanorods	Hydrothermal	2	−27.9	14.96	135
Fe_3_O_4_@SnO_2_ double shell	Hydrothermal	2	27.8	7	146
Fe@Al_2_O_3_	Mechanosynthesis	1.4	21.4	13.3	144
Spinel Fe_3_O_4_@TiO_2_ microsphere	Solvothermal, calcination	2	23.3	7	146
3D array Fe_3_O_4_@mesoporous C	Template assisted	2	−57	8	156
Fe@Al_2_O_3_/FeO_*x*_	Arc discharge	2.3	38.46	6.2	147
Fe nanoparticles@SnO_2_	Arc discharge	2	−39.2	16.8	148
Sr_0.8_La_0.2_Fe_11.8_Co_0.2_O_19_@Fe	Chemical vapor deposition	2.8	−30	8	157
Fe_3_O_4_@Cu-silicate	Sol–gel	2	−23	7	147
Fe_3_O_4_@PEDOT	Two step	2	−30	9.5	47
Fe_3_O_4_@PPy	Three step	2	−41.9	13.3	158
CeO_2_@Fe_3_O_4_	Two step solvothermal	—	−28.9	15.3	151
Durian like Fe_3_O_4_@TiO_2_	Solvothermal	2	−15.71	6.5	159
Fe nanoflake@SiO_2_	Ball milling	2.2–3.6	−20	3.8–7.3	145

**Ternary composites**					
ZnFe_2_O_4_@graphene/TiO_2_	Hydrothermal	2.5	55.6	3.8	160
ZnFe_2_O_4_@SiO_2_/RGO	Three step process	3.7	45.8	7.6	161
Fe_3_O_4_@SnO_2_/RGO	Three step process	4.5	45.5	6.4	162
HCNT/Fe@Fe_2_O_3_	Two step process	1.5	−45.8	8–9	163
Fe_3_O_4_/BaTiO_3_/RGO	Two step solvothermal	4	38.2	5	164
HGS@Fe_3_O_4_@RGO	Ferrite plating method	2.5	15.8	11	8
C@Fe@Fe_3_O_4_	Template, pyrolysis	1.5	40	5.2	165
Ag@Fe_3_O_4_/RGO	Solvothermal	2	40.05	11.9	154
G/Fe_3_O_4_@Fe	Multi steps process	2	−35.2	17–18	166
Fe/Fe_3_O_4_@C	IP	3.9	29.3	12.6	167
GN–pFe_3_O_4_@ZnO	Three steps process	5	−40	11.4	142
Fe@SiO_2_/PU	Surface initiated polymerization	1.8	−21.2	11.3	51
Fe@FeO/PU	Surface initiated polymerization	3	<−20	3.4	51
CIP@SiO_2_@Mn_0.6_Zn_0.4_Fe_2_O_4_	Co-precipitation	2	−44.24	11.57	168
Fe_3_O_4_@Fe/G	Two step process	4.6	−58	5.2	169
ZnFe_2_O_4_@RGO@CuS	Two step hydrothermal	2.2	−55.4	14.6	170

**Quaternary composites**					
Graphene@Fe_3_O_4_@WO_3_@PANI	Hydrothermal, chemical oxidation polymerization	4	46.7	9.4	171
G/Fe_3_O_4_@Fe/RGO	Multi steps process	2.5	−32.5	14–15	166
GNP/Fe_3_O_4_@BaTiO_3_/MWCNTs/VMQ	Solvothermal, solution blending	2.6	−26.7–33.3	1–20	172
G/Fe_3_O_4_@C@MnO_2_	Hydrothermal, thermal treatment	1.8	−38.8	15	173
ZnO/Fe/Fe_3_C/C	Thermal decomposition, heat treatment	1.5	−30.4	14.5	174
Graphene@Fe_3_O_4_@SiO_2_@PANI	Three steps process	2.5	−40.7	12.5	121
Dextran/Fe_3_O_4_@Fe/RGO	Solvothermal, hydrothermal	4	−20.26	4.72	175
Graphene@Fe_3_O_4_@PANI@TiO_2_	Hydrothermal, IP	1.6	−41	14.4	176
Epoxy–PPy/Fe_3_O_4_–ZnO	Co-precipitation, solution mixing	2	−32.53	9.96	177
Graphene@Fe_3_O_4_@PANI@TiO_2_	Hydrothermal, IP	1.6	−41	14.4	176
Polycarbonate/MWCNTs/Fe_3_O_4_@C	*In situ* hydrothermal	1	−41.3	17.7	178
Graphene@Fe_3_O_4_@SiO_2_@NiO	Hydrothermal, sol–gel	1.8	−51.5	14.6	179

##### 9.3.1.3 Dielectric core@magnetic shell

Another way of making a core@shell structure is to take dielectric materials as the core and magnetic materials as the shell. These materials can be chosen as we suggested in previous section. Wang *et al.*^151^ produced for the first time such a kind of core@shell structure using CeO_2_ (core) and Fe_3_O_4_ (shell). Their observations indicate that, compared to the magnetic@dielectric core–shell structure (Fe_3_O_4_@CeO_2_), the dielectric shell@magnetic core CeO_2_/Fe_3_O_4_ nanocapsules show improved dielectric properties owing to the increased O-vacancy concentration in the CeO_2_ cores of the larger grains as well as the O-vacancy-induced enhancement in interfacial polarization between the CeO_2_ cores and the Fe_3_O_4_ shells, respectively.

#### 9.3.2 Ternary composites

**Fig. 17:**
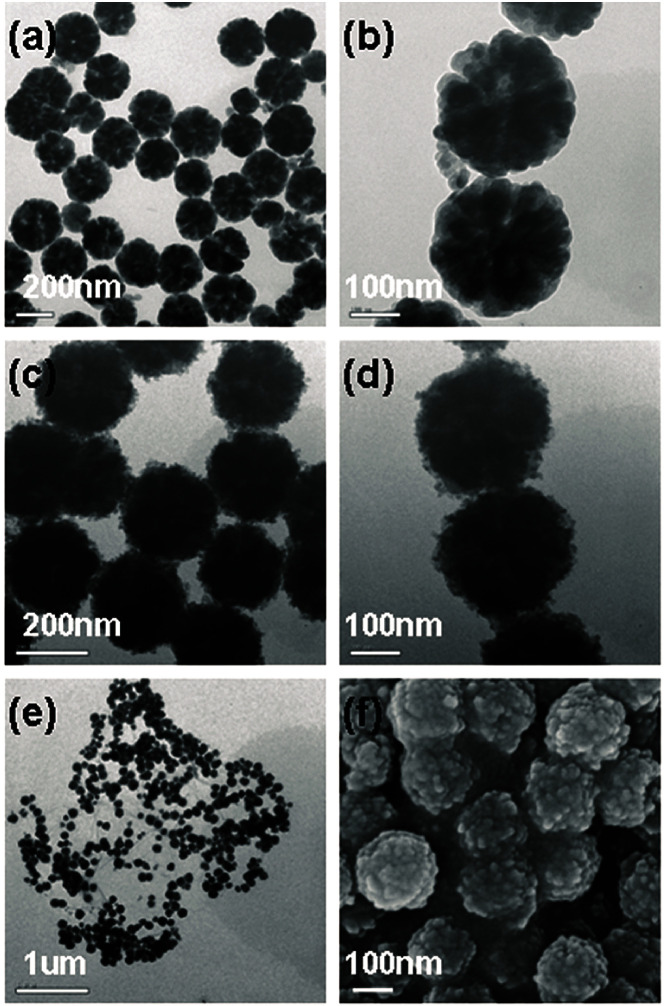
TEM images of the products at different stages (a and b) pFe_3_O_4_, (c and d) pFe_3_O_4_@ZnO, (e) GNpFe_3_O_4_@ZnO, and (f) corresponding FE-SEM image^181^ – reproduced by permission of the Royal Society of Chemistry.

**Fig. 18:**
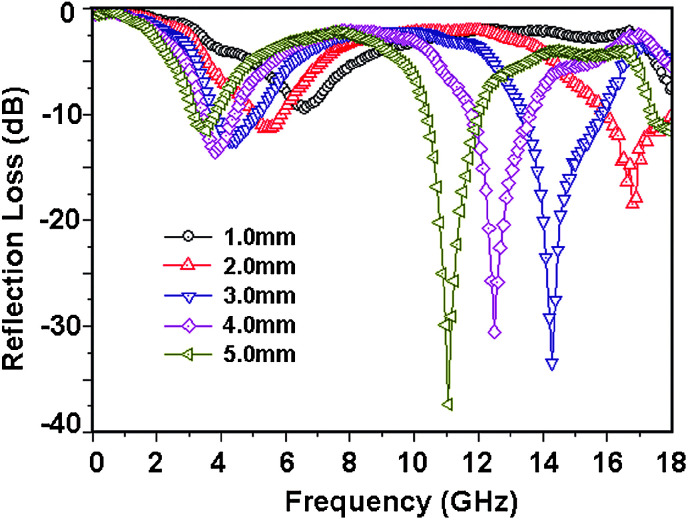
Reflection loss of GNpFe_3_O_4_@ZnO composites with thickness 1–5 mm  ^181^ – reproduced by permission of the Royal Society of Chemistry.

As we have seen in the binary composites section, the proliferation of microwave absorption performance is mainly an outcome of improved impedance matching, but we have neglected the important thing *i.e.* introduction of magnetic materials decreases the dielectric loss. This is a serious problem which influences the microwave absorption performance. Now, if we desire to preserve high dielectric loss even after inserting further magnetic materials, another high dielectric loss material must be introduced into the absorbing materials. For this purpose, carbonaceous materials such as graphene, CNTs, CFs or polymers like PANI, PPy *etc.* are preferred due to their attractive properties. In this case, the mixing of a magnetic material with a binary dielectric material not only enhances its attenuation ability, but also conserves the degree of impedance matching. In this direction, several composites such as Ag@Fe_3_O_4_/RGO,^154^ SiC@SiO_2_@Fe_3_O_4_,^180^ hollow carbon@Fe@Fe_3_O_4_ nanospheres^165^ and many others have been studied so for. Ternary composites can be made either by a core@shell@shell structure, by a combination of three materials or by dispersion of the core@shell structure into a matrix like graphene, PPy or PVDF *etc.* For example, Chen and co-workers produced a core@shell@shell-type structure from carbonyl iron powder (CIP)@SiO_2_@Mn_0.6_Zn_0.4_Fe_2_O_4_ ferrite. The as-prepared (CIP)@SiO_2_@Mn_0.6_Zn_0.4_Fe_2_O_4_ composites displayed better dielectric and magnetic loss characteristics at high frequency compared to pure CIP, CIP@SiO_2_ and CIP@Mn_0.6_Zn_0.4_Fe_2_O_4_.^168^ Sun and coworkers^181^ prepared mesoporous Fe_3_O_4_@ZnO sphere decorated graphene (GN-pFe_3_O_4_@ZnO) composites with sufficient porosity, uniform size, high magnetization and excellent EM wave absorption properties. They adopted a three-step method to synthesise these composites. The as-prepared pFe_3_O_4_ nanoparticles have a mean diameter of 200 nm (Fig. 17a and b), but coating the ZnO layer increases the diameter of the pFe_3_O_4_@ZnO spheres (Fig. 17c and d). Fig. 17e and f represent the TEM image of pFe_3_O_4_@ZnO sphere decorated by graphene (GN). It is evident that each GN sheet is well anchored by pFe_3_O_4_@ZnO spheres and no individual pFe_3_O_4_@ZnO sphere can be observed outside of the GN sheet. The EM wave absorption properties of the GN-pFe_3_O_4_@ZnO composites were studied in the thickness range of 1–5 mm. A stronger RL peak is found at high-frequency and also at the normal RL peak. With increasing thickness, both peaks shift from the high frequency to the low-frequency side and show a decreasing minimal RL value (Fig. 18). The minimal RL of the GN-pF_3_O_4_@ZnO composite was almost −40 dB, with an absorption bandwidth corresponding to RL < −10 dB at 11.4 GHz frequency.

#### 9.3.3 Quaternary composites

Nowadays researchers have focused on the synthesis of quaternary composites. The benefit of quaternary composites over ternary composites is the presence of multiple interfaces, as we know that interfacial polarization plays a crucial role in EMI-preventing materials. Therefore, multiple interfaces in heterogeneous quaternary composites not only enhance the dielectric loss due to interfacial and space polarization but also promote multiple reflection owing to their complicated morphologies. These can be synthesized either in core@shell@shell hetero nanostructures distributed on surface of substrates like graphite, graphene, CNTs, PANI *etc.* or by combinations of different materials. Wang and coworkers^121^ fabricated core@shell@shell/substrate type Fe_3_O_4_@SiO_2_@polyaniline hetero nanostructures wrapped with a graphene substrate. According to them, the presence of triple-interfaces and junctions in the Fe_3_O_4_@SiO_2_@polyaniline/graphene composites increases the interfacial polarization. Meanwhile, active sites present in PANI and the void space between Fe_3_O_4_ and PANI led to a somewhat large specific surface area which increases the reflection of EM waves. The above-moentioned void spaces are effective in terms of restricting the spreading of EM waves and produce heating because of the impendence dissimilarity, boosting the microwave absorption properties. Although quaternary composites nevertheless have several benefits, the synthesis of quaternary core–shell is quite complicated and hence they have been the subject of few studies.

### 9.4 Fe species with 1D, 2D and 3D carbon materials

The third strategy is to assemble magnetic Fe-related nanomaterials onto one-dimensional 1D and 2D carbon materials like CNTs, CNFs, 2D graphene or 3D graphene sheets, or graphene capsules to form a hybrid structure. Carbonaceous materials are a high-dielectric material. However, these materials cannot be used alone due to their limitations in terms of impedance matching with the absorber matrix. Thus the combination with magnetic materials is beneficial to improve the microwave absorption, due to their adjustable dielectric and magnetic properties. He *et al.* observed superior performance in flaky carbonyl iron (FCI) coated with RGO nanosheets. In fact, FCI/RGO is a typical example of the dielectric dispersion behaviour of complex permittivity in which the contribution of RGO as a dielectric lossy material confers FCI with advanced dielectric loss and magnetic loss abilities.^66^ Hu *et al.*^182^ have shown the remarkable microwave absorption properties of 3D reduced graphene oxide and single-crystalline Fe_3_O_4_. In 3D graphene/Fe_3_O_4_ composites, graphene confers a big contact surface for the homogeneous distribution of Fe_3_O_4_ particles because 3D graphene has a large surface area compared to 2D graphene. Therefore, 3D graphene acts as an ideal substrate for the absorption of microwaves. This improves both the dielectric loss and magnetic loss, hence the improved absorption characteristics for the 3D graphene/Fe_3_O_4_ composites in comparison to those of the 3D graphene and Fe_3_O_4_. On other hand, to explore the probable attenuation process in trilayer MnO_2_@Fe-graphene composites, an antenna mechanism of the rod-like structure was proposed by Lv and coworkers. According to them, EM energy transfers in the form of a microcurrent in the rod-like structures. Furthermore, addition of Fe with MnO_2_ increases impedance matching so that MnO_2_@Fe composite converts more-and-more EM energy into the microcurrent. This microwave current is now expected to transmit from one rod to another. In these condition, the graphene works as an electrically conductive network path, since electrical energy is attenuated due to the resistance of the graphene. Meanwhile, dual interfaces *e.g.* Fe–C and MnO_2_–Fe also lead to electron polarization, carrying through the attenuation EM wave.^5^ Many examples of ternary (*e.g.* PANI/graphene@Fe_3_O_4_), quaternary (*e.g.* graphene@Fe_3_O_4_@WO_3_@PANI) and even quinary composites (*e.g.* methyl vinyl silicone rubber (VMQ)-graphene nanoplate/Fe_3_O_4_@BaTiO_3_/MWCNTs) can be seen on graphene, which is one of the most important substituents.^171,172^ To illustrate this, we shall take the example of the quaternary composite Fe_3_O_4_@BaTiO_3_/MWCNTs. In this composite, firstly the presence of multi-interfaces causes the enhancement of interfacial polarization. Secondly, the layering of PANI on the graphene sheet is attributed to electron tunnelling and the development of electronic clouds which are responsible for converting EM energy into heat energy. It is a well-known fact that EM wave absorption properties depend significantly on the microstructure of the absorbers. Once the EM wave strikes the absorbers, the sandwich multilayer structure can efficiently enhance multiple reflection and offer a maximum absorption of the EM wave and a minimum reflection. In addition, the upturn in absorption also causes better impedance matching and a synergistic effect. This accounts for the superior absorption properties of graphene@Fe_3_O_4_@WO_3_@PANI composites. Some popular examples of multi-functional carbon materials with Fe components are listed in Table 4.

**Table 4:** Fe component/carbon based microwave absorber

Carbonaceous based Fe composites					
Materials	Method	Thickness/mm	RL_min_/dB	Frequency/GHz	Ref.
CNT/Fe_3_O_4_/RGO	Solvothermal, ultrasonic method	2.5	−50.0	8.7	30
RGO/Fe_3_O_4_	Hummer, solvothermal	2	−27	5.4	183
Bowl like Fe_3_O_4_/RGO	Solvothermal	2	−24	12.9	184
Fe_3_O_4_/RGO composites	One-pot co-precipitation	3.9	−44.6	6.6	185
NiFe_2_O_4_ nanorod/graphene	One-step hydrothermal	2	−29.2	16.1	186
Popcorn likeα-Fe_2_O_3_/3D G	Template assisted, co-precipitation	1.4	−55.7	17.3	42
α-Fe_2_O_3_ nanorod/graphene	Chemical reduction	2	−45	12.8	187
Flaky CI/RGO	Modified Hummer, reflux	3.87	−64.4	5.2	66
RGO/α-Fe_2_O_3_ hydrogel	Two-step hydrothermal	3	−33.5	0.712	44
RGO/spherical CI	Wet chemical method	3	−52.46	7.79–11.98	188
RGO/NiFe_2_O_4_	One-step hydrothermal	3.0	−39.7	9.2	189
RGO/CoFe_2_O_4_ZnS	Hydrothermal, co-precipitation	1.8	−43.2	10.2–15.7	190
RGO/CoFe_2_O_4_SnO_2_	Two steps hydrothermal	1.6	−54.4	16.5	191
Fe_3_O_4_//graphene	Two steps	1.5	−29	8–12	81
α-Fe_2_O_3_/γ-Fe_2_O_3_/RGO	Thermochemical reactions	4.0	−13.6	3.76	43
ZnFe_2_O_4_@graphene@TiO_2_	Hydrothermal	2.5	−55.6	3.8	160
TiO_2_/RGO/Fe_2_O_3_	Hydrothermal	2.0	−44.0	14.8	192
RGO/SiO_2_/Fe_3_O_4_	Two steps reaction	4.5	−56.4	8.1	193
RGO/ZnFe_2_O_4_	Solvothermal	2.5	−41.1	9.4	194
Co_0.5_Ni_0.5_Fe_3_O_4_/RGO	Chemical reduction	2.5	−13.1	14.8	195
Ni_0.8_Zn_0.2_Ce_0.06_Fe_1.94_O_4_/GNS	Sol–gel deoxidation technique	—	−37.4	12.3	196
RGO/BaFe_12_O_19_/Fe_3_O_4_	Two step hydrothermal	1.8	−46.04	15.6	197
RGO@Fe_3_O_4_	Two step process	2.0	−56.25	12.62	198
Porous Fe_3_O_4_/C	Hydrothermal process	3	−31.75	7.76–12.88	199
Fe–C nanocapsules	Arc-discharge method	3.1	−43.1	9.6	200
Fe_3_O_4_C yolk–shell	*In situ* reduction process	3	−45.8	10.6	152
Fe_3_O_4_ microsphere@C	*In situ* polymerization	2	−20.6	13.4	23
CI/C	Hydrothermal	1.3	−46.69	11.5	201
Fe_3_O_4_ graphite	Molten salt route, temperature reduction	4.8	−51	4.3	202
EG/Fe_3_O_4_	Solvothermal, sintering route	2.6	−24.8	6.8	203
EG/Fe/Fe_3_O_4_	Chemical vapor deposition	1.9	−42.4	9.36	204
Epoxy graphitized nanosheet@Fe_3_O_4_–MnO_2_	Hydrothermal	4.5	−31.7	5.85	205
Flower like Fe_3_O_4_/MWCNTs	Acid treatment, hydrothermal	0.9	−64*	18	206
CNTs/Fe_3_O_4_/RGO	Solvothermal, ultrasonic method	2.5	−50.0	8.7	30
CNTs/CoFe_2_O_4_	Chemical vapor deposition	—	18	9	3
MWCNTs/Fe_2_O_4_	*In situ* growth method	1.5	−30	5.7	207
MWCNT/NiO–Fe_2_O_4_	Electroless plating	2	−55	3.5–18	208
Fe_2_O_3_/Fe_3_O_4_/MWCNTs	Hydrothermal	2.5	44.1	10.4	209
Fe-MWCNT	Chemical method	4.27	−39	2.7	210
Fe_3_O_4_/CF	Electrophoretic deposition	1.7	−11	10.37–11.4	211

### 9.5 Fe ingredients with polymers

In general, different methods have been adopted to coat metal films onto a substrate (another material) for shielding purposes. Nonetheless the poor scratch resistance and enrobing weaken their application to a certain extent. In this aspect, an electrically conducting polymer matrix either with a magnetic filler or without a filler can be a good alternative to metals. It is important that the crucial loss mechanisms in non-magnetic materials (like carbon materials and conductive polymers) are the dielectric (dipolar) and conduction losses. Conduction losses typically dominate in high conductivity materials and dipolar losses dominate in poor conductivity materials. It was seen that pure conducting polymers weakly absorb the EM wave, as Zhang *et al.* observed for pure PANI, in which RL_min_ reached only −18 dB at 13.8 GHz with a thickness of 2 mm, which shows PANI weakly absorb EM waves; whereas Sui *et al.* have reported a similar result obtained for PPy, which possesses an RL_max_ of 16.7 dB at 17.6 GHz. Another conducting polymer, pure PEDOT, also has weak attenuation towards EM waves, and has an RL_max_ of only −14.5 dB at 7 GHz, as investigated by Zhang *et al.*^260–263^ On other hand, magnetic materials show magnetic losses such as hysteresis, electron spin resonance and domain wall resonance *etc.* Additionally, magnetic materials with relatively higher electrical conductivity exhibit great conduction losses. Hence, effective EMI shielding can be achieved when materials exhibit both magnetic and dielectric loss processes together. In this context, ferrites and iron oxides are extensively used for the improvement of the electrical as well magnetic properties of conductive polymers. Liu and coworkers^219^ made barium hexa-ferrite (BaFe_12_O_19_)@PANI core@shell nano-composites. Addition of BaFe_12_O_19_ benefits the conductance loss and magnetic resonance loss as well interfacial loss. Moreover, tuning the shell thickness provides us with optimal impedance matching. As can be seen in BaFe_12_O_19_@PANI composites, the maximum absorption loss was 28 dB at 12.8 GHz with an absorption bandwidth of 3.8 GHz and a thickness of 2.0 mm (30–40 nm thick shell). Moreover, tuning the mole ratio of the doped acid to the monomer may give a non-magnetic state (NM). This NM state weakens the dielectric loss and magnetic loss simultaneously, but raises the impedance matching and establishes the complementary behaviour between the dielectric loss and the magnetic loss, and strengthens the absorbing properties of the composites. Fan and coworkers investigated the effect of different acid [*p*-toluenesulfonic acid]/[aniline] ratios ([*p*-TSA]/[ANI] = 0.005/1, 0.05/1, 0.2/1) on the microwave properties of PANI/CIP composites.^264^ Among all the ratios, 0.2/1 shows the best reflection loss (>−20 dB) due to the appearance of a nonmagnetic state owing to the CIP size (microspheres). On other hand, the core–shell structure of CIP@PANI composites suppresses saturation magnetization, weakening the magnetic loss and dielectric loss, but enhancing impedance matching. Nowadays, carbon materials are also being used with conducting polymer and iron materials because the carbon material not only serves as superior substrate but also improves the mechanical and thermal characteristic of these composites. Besides, carbon materials facilitate the hopping of charge carriers, enhance multiple reflection and increase the interfacial polarization. Considering all these points, Wang *et al.* for the first time fabricated FeCo@SnO_2_@graphene@PANI quaternary composites with three SnO_2_, graphene and PANI dielectric loss absorbers, while the FeCo particles serve as magnetic loss absorbers. A maximum reflection loss was reached of −39.8 dB at 6.4 GHz with a thickness of 3 mm due to the high specific surface area and the presence of residual defects and organic groups of RGO that act as polarized centers, increasing the polarization relaxation process and multiple reflections. Moreover, hopping charge carriers enhance the eddy current loss between PANI and graphene which converts electrical energy into heat energy. This was despite the fact that multi-interfaces between FeCo, SnO_2_, PANI and graphene work as polarization centers and create dipole and interfacial polarization of the composites owing to the synergistic effects of different types of material.^124^ Some of the studied polymer composites are listed in Table 5. Therefore, Fe materials are widely used with these polymers. But then again, these polymers suffer limitations because of their poor processibility and mechanical properties. Therefore, rubber polymer composites containing Fe-based fillers have also been examined as effective EMI shielding materials because of their unique combination of polymeric flexibility, electrical conductivity and magnetic properties, as reported by Nasouri and coworkers.^258^ This group used Fe_3_O_4_ as nanofiller in a polyvinylpyrrolidone (PVP) matrix. It was found that with increasing Fe_3_O_4_ nanoparticle concentration EMI shielding efficiency increases up to 22 dB (4% of Fe_3_O_4_ filler) in which absorption was the major shielding mechanism. Similarly, Al-Ghamdi *et al.* used Fe_3_O_4_ as filler in an NBR matrix^257^ and obtained an 80–90 dB SE_T_ at 40% of Fe_3_O_4_ filler. For moderate conductivity Fe materials, such as Fe_3_O_4_, a large quantity of nanofiller is required to reach the threshold value to connect the conducting path in insulating matrices. Thus, to enhance the further electrical conductivity, the concept of double percolation can be adopted by means of using RGO, CNTs, black carbon *etc.* as reported by Pawar *et al.*^259^ in MWCNT/Fe_3_O_4_ within PC (polycarbonate)/SAN [poly(styrene-*co*-acrylonitrile)] blend composites, in which an SE_T_ of around −32.5* dB at 18 GHz was observed for 3 wt%-MWCNT and 3 vol%-Fe_3_O_4_ in 60/40 PC/SAN blends. In this case, absorption occurs due to synergy between the MWCNTs and Fe_3_O_4_ nanoparticles in which the MWCNTs absorb the electrical field while the dopamine-anchored Fe_3_O_4_ absorbs the magnetic field of the EM radiation, resulting in improved EMI shielding.

**Table 5:** Polymer and Fe materials based microwave absorber

Polymer based composites					
Materials	Methods	Thickness/mm	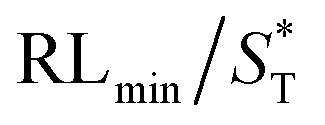 /dB	Frequency/GHz	Ref.
PANI@Fe_3_O_4_ hybrid	Solvothermal, IP	4.5	29.3	7	212
PANI/Fe_3_O_4_	*In situ* polymerization (IP)	0.5	−54	33.72	213
Hollow PANI/Fe_3_O_4_	Three steps process	2	−10	9–10	214
Fe_3_O_4_@PANI NPs	Oxidation reduction, IP	1.7	35.1	16.7	215
Fe_3_O_4_@PANI MS	IP	2	−37.4	15.4	216
HPAP/Fe_3_O_4_	Co-precipitation, *in situ* EP	2	−3	9–10	217
PANI/BaFe_12_O_19_	IP	2	−12	32.3	218
BaFe_12_O_19_@PANI	Sol–gel, auto-combustion, IP	2	−28	12.8	219
PANI/MWCNTs/Fe_3_O_4_	Co-precipitation, IP	4	16	8–15	220
PANI@nano-Fe_3_O_4_@CFs	Three steps process	1.5	−11.11	8.1–18	221
PANI@graphene@Fe_3_O_4_	Hydrothermal, IP	3	−43.7	10.7	222
PANI/Zn_0.6_Cu_0.4_Cr_0.5_Fe_1.46_Sm_0.04_O_4_	Rheological phase reaction, IP	2	−22.46	2–18	223
PANI/CIP/Fe_3_O_4_	Co-precipitation, *in situ* EP	1.76	48.3	9.6	224
PANI/CuI/Fe_3_O_4_	Mechanical mixing	2.8	35.3	13.28	225
PANI/PPy/Fe_3_O_4_	Three steps	2	−47.3	13.45	226
PANI/Ag/SrFe_12_O_19_	Three steps	3	−14.86	9.98	227
PANI/MnFe_3_O_4_	Three steps	1.4	−15.3	10.4	228
Graphene@Fe_3_O_4_@SiO_2_@PANI	Stöber method, IP	2.5	−40.7	12.5	121
PANI–BF_0.25_	Surfactant assisted solvothermal, IP	2.5	−45.2	11.2	229
CuS/RGO/PANI/Fe_3_O_4_	Hydrothermal, IP	2.5	−69.2	10.2	222
Fe_3_O_4_@PPy microspheres	Chemical oxidative polymerization	2.5	−31.5	15.5	230
PPy/Fe_3_O_4_/PVDF	Chemical vapor deposition, IP	2.5	−21.5	16.8	54
PPy/Zn_0.6_Cu_0.4_Cr_0.5_Fe_1.46_Sm_0.04_O_4_	Rheological phase reaction, IP	2	−20.9	14.05	223
PPy–γ-Fe_2_O_3_ fly ash	*In situ* emulsion polymerization (EP)	2	25.5*	12.4–18	223
Fe_3_O_4_@SiO_2_@PPy	Microemulsion polymerization	5	40.9	6	231
γ-Fe_2_O_3_/(SiO_2_)_*x*_SO_3_H/polypyrrole	Solgel, IP	4	43.1	15.1	46
γ-Fe_2_O_3_–SiO_2_–PEDOT	Two step reaction	2	−27.5	13.8	232
Hollow γ-Fe_2_O_3_@SiO_2_@PEDOT	Two step reaction	2	−21.3	14.1	233
Fe_3_O_4_/PEDOT	Mechanical mixing	4	−15.8	3.2	234
BaFe_12_O_19_/PEDOT	*In situ* EP	—	24.5*	15	235
γ-Fe_2_O_3_@PEDOT	Two step reaction	2	−44.7	12.9	233
Fe_3_O_4_–RGO/PIL–PEDOT	Poly(ionic liquid) mediated hybridization	0.01	22*	0.02–1	236
Fe_3_O_4_–PEDOT nanospindles	Oxidative molecular layer deposition	1.4	−55	16.2	237
PEDOT:PSS/Fe_3_O_4_	Reflux mixing	—	40*	8–12.5	238
Fe_3_O_4_/C/PVDF	Wet chemical method, heat treatment	2.1	−38.8	11–12	239
PVDF/Fe_3_O_4_/CNT	Twin screw compounding method	0.7	28.8	5.6	240
PVDF/Fe_3_O_4_–PANI/SWCNH	IP, solution blending	2	−29.7*	14.5–20	241
Fe_3_O_4_/PVDF/PPy	Chemical vapour deposition, IP	2.5	−21.5	16.8	54
PVDF/PS/HDPE/MWCNT/Fe_3_O_4_	Melt blending	—	25*	9.5	242
Flake shaped CI/RGO/PVP	Chemical reduction	1.5	−130.3	16.88	52
Fe_3_O_4_/PEI	Co-precipitation	2.4	−30.69	7.24	243
PS/graphene/Fe_3_O_4_	Solution blending	—	30*	9.8–12	244
PVA–GAPC–Fe_3_O_4_	Solution casting method	0.3	15*	8.3–12.4	245
FePc–Fe_3_O_4_–basalt fiber	Solvothermal	5	−31.1	5.9	246
Flake carbonyl-Fe/MAA/PS	Dispersion polymerization	2.5	−39	3.3	247
Ni_0.5_Zn_0.5_–Fe_2_O_4_/PU	Mixing method	3	−2.8	15–16	248
CoFe_2_O_4_/paraffin	Surfactant-assisted hydrothermal	2	−40	10.7	249
CoFe_2_O_4_/epoxy	Surfactant-assisted hydrothermal	2	−59.8	11.86	249
EVA/polycrystalline iron fibers	Mechanical method	2.0	23.7	7.2	250
CI/polyurethane	Mechanical mixing	3	−25.2	6.64	251
CI/epoxy-silicone	Mechanical mixing	2.5	−40	6.2	252
Flaked shape CI/RGO-epoxy	Ball milling	2	−32.3	11	253
CI/Ethylene propylene diene monomer rubber (EPDM)	Thermal decomposition, two roller mixer	3	−21.7	3.5	59
Epoxy/silicon/GNS/flake CI	Multi-step process	1.2	−8	6.6–18	254
Fe/silicone rubber	Mixing process	1	−5	1–2	255
Polydimethylsiloxane (PDMS)/CNFs/Fe_3_O_4_	Solution-casting	5.5	44	15.75	256
Nitrile butadiene rubber (NBR)/Fe_3_O_4_	Hydrothermal, two roller mixing	2	80–90*	1–12	257
Polyvinyl pyrrolidone (PVP)/Fe_3_O_4_	Two step process	1	22*	8.2–12.4	258
Polyvinyl chloride (PVC) graphene/Fe_3_O_4_	Co-precipitation, heating	1.8	13*	8–12	61
MWCNT/g-Fe_3_O_4_ polycarbonate/poly (styrene-*co*-acrylonitrile)	Three step process	—	32*	18	259

## 10 Conclusions

In brief, iron (Fe) and its oxide materials are very useful from an applications points of view in the fields of energy, medical, research and many others. In this review paper, we explored composites comprising carbonaceous, polymer and dielectric materials with iron components as important constituents for the prevention of electromagnetic interference (EMI) by reflection as well as by absorption. Two losses, dielectric and magnetic, are responsible for high microwave absorption and the total shielding performance. In this context, iron and its components can be versatile choice for EMI shielding applications in combination with conductive polymers and carbon materials *etc.* which integrate with its EMI efficiency.

## Conflicts of interest

There are no conflicts to declare.

## Supplementary Material
